# Reducing undesired solubility of squarephaneic tetraimide for use as an organic battery electrode material†

**DOI:** 10.1039/d3fd00145h

**Published:** 2023-08-17

**Authors:** Bowen Ding, Manik Bhosale, Troy L. R. Bennett, Martin Heeney, Felix Plasser, Birgit Esser, Florian Glöcklhofer

**Affiliations:** a Department of Chemistry and Centre for Processable Electronics, Imperial College London, Molecular Sciences Research Hub (White City Campus) 80 Wood Lane Shepherd's Bush London W12 0BZ UK f.glocklhofer@imperial.ac.uk; b Institute of Organic Chemistry II and Advanced Materials, Ulm University Albert-Einstein-Allee 11 89081 Ulm Germany birgit.esser@uni-ulm.de; c Physical Sciences and Engineering Division (PSE), King Abdullah University of Science and Technology (KAUST) Thuwal 23955-6900 Saudi Arabia; d Department of Chemistry, Loughborough University Loughborough LE11 3TU UK; e Institute of Applied Synthetic Chemistry, TU Wien Getreidemarkt 9/163 1060 Vienna Austria florian.gloecklhofer@tuwien.ac.at

## Abstract

Locally aromatic alkyl-*N*-substituted squarephaneic tetraimide (SqTI) conjugated macrocycles are four-electron reducible, owing to global aromaticity and presumed global Baird aromaticity of the dianion and tetraanion states, respectively. However, their good solubility inhibits their application as a battery electrode material. By applying sidechain removal as a strategy to reduce SqTI solubility, we report the development of its unsubstituted derivative SqTI-H, which was obtained directly from squarephaneic tetraanhydride by facile treatment with hexamethyldisilazane and MeOH. Compared to alkyl-*N*-substituted SqTI-Rs, SqTI-H exhibited further improved thermal stability and low neutral state solubility in most common organic solvents, owing to computationally demonstrated hydrogen-bonding capabilities emanating from each imide position on SqTI-H. Reversible solid state electrochemical reduction of SqTI-H to the globally aromatic dianion state was also observed at −1.25 V *vs.* Fc/Fc^+^, which could be further reduced in two stages. Preliminary testing of SqTI-H in composite electrodes for lithium–organic half cells uncovered imperfect cycling performance, which may be explained by persistent solubility of reduced states, necessitating further optimisation of electrode fabrication procedures to attain maximum performance.

## Abstract 2

Unsubstituted squarephaneic tetraimide with reduced solubility due to H-bonding interactions is synthesised, characterised, and tested as a battery electrode material.

## Graphical abstract

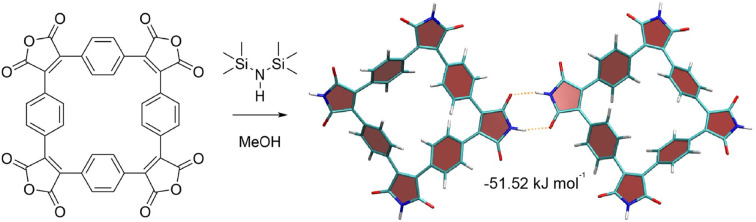

## Introduction

**Scheme 1:**
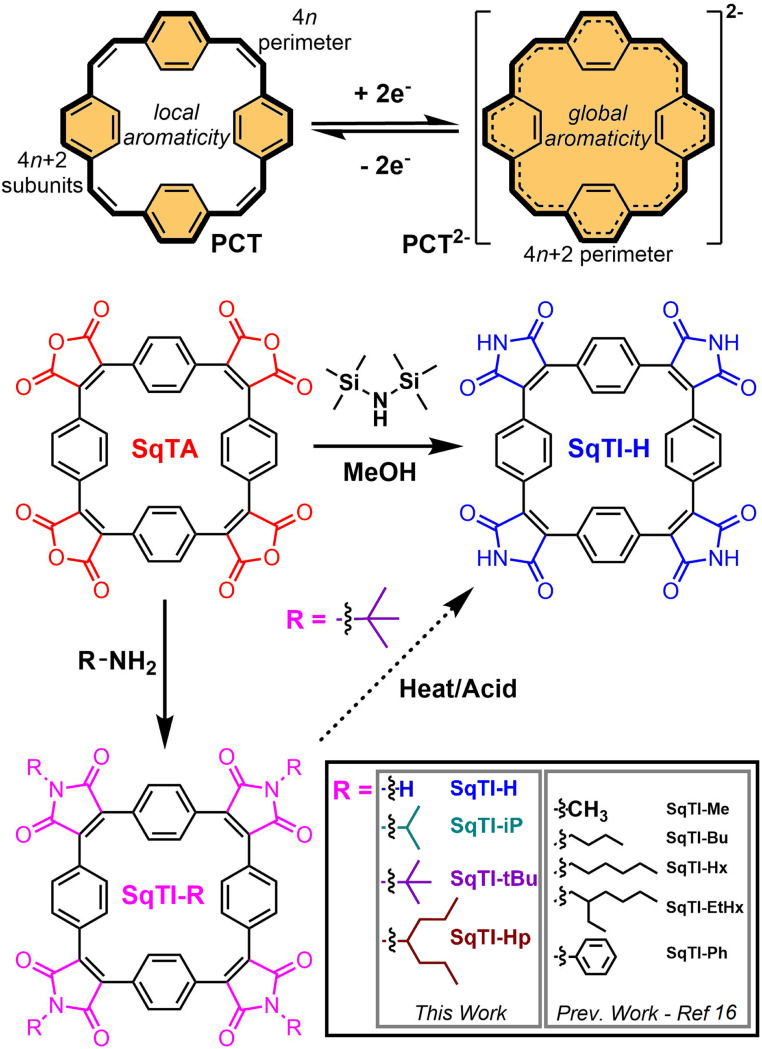
Chemical structures of paracyclophanetetraene (PCT, top), including the redox switching of PCT from the locally aromatic neutral state (with concealed antiaromaticity) to the globally aromatic dianion state, as well as squarephaneic tetraanhydride (SqTA, middle left), including conversion of SqTA to various substituted squarephaneic tetraimides (SqTI-Rs, bottom) and unsubstituted SqTI-H (middle right) reported herein.

Organic redox-active compounds have captured the attention of recent research into energy storage, owing to their molecular tunability, which facilitates their application-specific optimisation.^1–4^ Within the conglomeration of organic redox-active materials, conjugated macrocycles exhibit particularly functional attributes, combining robust redox characteristics enabled by their protracted architectures with excellent reproducibility that can only be achieved with discrete molecular systems.^5–7^ [2.2.2.2]Paracyclophane-1,9,17,25-tetraene (PCT, Scheme 1, top) is one particular conjugated macrocycle that exemplifies this type of organic redox-active materials,^8^ owing to its predictable synthesis and reversible two-electron electrochemical reduction. The reversible reduction of PCT is enabled by its type I concealed antiaromaticity; in the neutral state, locally aromatic phenylene units conceal the antiaromaticity of the formal macrocyclic conjugated system with 4*n* (24) π electrons that drives excellent stability of the globally aromatic dianion state with 4*n* + 2 (26) π electrons.^9^ Similar aromaticity switching also plays a role in other reported organic battery electrode materials.^10–13^ For PCT, the reversible two-electron electrochemical reduction, in combination with porosity for lithium and sodium ions, determined crystallographically at ∼14–17% and ∼4–5% v/v respectively (depending on phase), enable its application as a battery electrode material. However, in common battery electrolytes, PCT cannot be reduced beyond the dianion state, which limits its specific capacity, whilst the increased solubility of the dianion state hampers its cycling performance, if not addressed by the appropriate choice of conductive agent and binder.

In order to raise specific capacity and, drawing inspiration from the uniquely stable redox properties of aromatic cyclic anhydrides and their imide derivatives, such as naphthalenediimide,^14,15^ our focus has shifted towards the development of squarephaneic tetraanhydride (SqTA) and its tetraimide derivatives (SqTI, Scheme 1),^16^ which builds on the porous PCT substructure with switching between different locally and globally aromatic states. Our development of SqTA opened up its conversion to a number of alkyl-*N*-substituted squarephaneic tetraimides (SqTI-Rs),^16^ which featured reversible access to the four-electron reduced state, owing to global aromaticity of the dianion and presumed global Baird aromaticity of the triplet tetraanion.^17,18^ However, alkyl sidechain presence in these SqTI-Rs confers excellent solubilities, which significantly hinders their physical stability upon cycling as battery electrode materials. Therefore, strategies that develop upon the molecular design of the four-electron reducible SqTI unit for applications in energy storage need to focus on minimising solubility.

Generally, compound solubility can be reduced by strengthening intermolecular interactions, for example π–π interactions, or minimising favourable solvent interactions, through omitting sidechains. Conjugation extension of SqTI to increase π–π interactions may be achieved through its conversion into derivatives suitable for subsequent cross-coupling functionalisation,^19^ or by its potential incorporation into covalent-organic frameworks^20–24^ where its *D*_4_ symmetry may be exploited to template reticular framework synthesis^25^ by lattice propagation at the four anhydride positions of SqTA.^26^ However, specific capacity is lowered if the molecular weight per redox-active unit is increased by conjugation extension. On the other hand, SqTI featuring unsubstituted protonated imides, giving sidechain omitting SqTI-H (Scheme 1), also introduces opportunities for intermolecular hydrogen-bonding interactions at the imides that further strengthen solid state packing. The thermal cleavage of branched sidechains on alkyl-*N*-substituted SqTI-Rs has been identified as a possible route to SqTI-H, which was reported previously for perylenediimide incorporated into two-dimensional conjugated polymers.^27,28^ Acidic cleavage of cyclic amide *tert*-butyl sidechains in neat fluorinated strong acids has also been reported,^29,30^ and has been identified as a potential alternative to thermal cleavage at the imide of SqTI-Rs. Direct conversion of SqTA to SqTI-H may also be possible, through the treatment of SqTA with hexamethyldisilazane (HMDS) and MeOH, which mixes *in situ* to generate ammonia and methoxytrimethylsilane, the latter of which activates the amic acid intermediate by converting it to the silyl ester, propelling formation of the unsubstituted imide under mild conditions.^31–33^

Herein, we report our exploration into the synthesis of SqTI-H from SqTA *via* the aforementioned three routes. The direct conversion of SqTA to SqTI-H with HMDS and MeOH at room temperature was found to be most effective, yielding SqTI-H in ∼70% yield. Physical characterisations revealed as-synthesised SqTI-H exhibits significantly reduced solubility and further improved thermal stability compared to alkyl-*N*-substituted SqTI-Rs, owing to the hydrogen-bonding capabilities emanating from each of the four unsubstituted imide positions, as deduced computationally. Solid state electrochemistry studies of locally aromatic SqTI-H revealed its two-electron reduction centred about −1.25 V *vs.* Fc/Fc^+^ to reach the globally aromatic dianion state, which can be further reduced in two one-electron reduction stages to form the trianion and tetraanion species. Preliminary testing of SqTI-H as an electrode material within lithium–organic half cells revealed imperfect cycling performance, requiring further electrode fabrication optimisation in order to consistently access peak specific capacity.

## Results and discussion

For the acidic and thermal cleavage routes, SqTI-Rs featuring isopropyl, *tert*-butyl and 4-heptyl groups at each of the four imide positions on SqTI, giving SqTI-iP, SqTI-tBu and SqTI-Hp respectively (Scheme 1), were synthesised from SqTA according to a modified procedure from that reported previously for the synthesis of similar alkyl-*N*-substituted SqTI-Rs.^16^ THF was employed as the solvent in place of glacial acetic acid, alongside conventional overnight heating *in lieu* of microwave heating, to give good yields ranging from ∼50–80%, demonstrating compatibility of SqTA conversion to alkyl-*N*-substituted SqTI-Rs with various synthetic conditions. Chemical structures of SqTI-iP, SqTI-tBu and SqTI-Hp were confirmed through their NMR spectra (Fig. S1–S7†) and high-resolution mass spectrometry (HRMS, Fig. S11–S13†).

The cleavage of *tert*-butyl groups on *N*-substituted cyclic amides has been previously reported to occur at room temperature in neat trifluoromethanesulfonic acid and at reflux in neat trifluoroacetic acid,^29,30^ whilst cleavage of *tert*-butyl groups on *N*-substituted carbamates is known to occur with catalytic amounts of trifluoromethanesulfonic acid.^34^ Drawing inspiration from these studies, the acidic cleavage of SqTI-tBu was attempted in our study by refluxing overnight in trifluoroacetic acid. Subsequent removal of reaction solvent and direct ^1^H NMR analysis (Fig. S8†) revealed multiple peaks between 7.53–7.51 ppm assigned to the aromatic protons about the SqTI macrocycle, indicating incomplete conversion. However, there was no observed variability in the positions of singlet signals assigned to (uncleaved) *tert*-butyl and (cleaved) imide protons, at 1.60 and 11.38 ppm, respectively, allowing their integration analysis to determine occurrence of an overall ∼70% cleavage of *tert*-butyl sidechains. With further optimisation, the complete acidic cleavage of SqTI-tBu may be possible to give SqTI-H.

In order to examine the possibility of SqTI-iP, SqTI-tBu and/or SqTI-Hp conversion to SqTI-H by thermal sidechain cleavage, their thermogravimetric analysis (TGA, Fig. S14†) was conducted. No evidence of mass loss stages corresponding to the formation of SqTI-H were observed in the TGA traces of SqTI-iP and SqTI-Hp, only pyrolysis above 450 °C (Fig. S14c and d†). The TGA trace of SqTI-tBu (Fig. S14b†) revealed a gradual initial mass loss of ∼25% upon heating to 260 °C, which may correspond with loss of four *tert*-butyl sidechains according to mass calculations. However, this thermolysis product was observed to be only *quasi*-stable up to 340 °C; increased heating uncovered a second mass loss stage, not observed later in the TGA of directly synthesised SqTI-H (Fig. S14a†), suggesting thermal sidechain cleavage of SqTI-tBu is unlikely to be a reliable synthetic route to SqTI-H.

Ultimately, the most effective pathway to SqTI-H was found to be its direct conversion from SqTA with HMDS/MeOH, according to conditions first reported by Davis and co-workers for the conversion of maleic anhydrides to imides.^31^ Addition of HMDS and MeOH into the reaction generates ammonia and methoxytrimethylsilane *in situ*, which converts the maleic anhydride group to the silyl ester amic acid intermediate, which is activated towards its re-cyclisation to form the corresponding imide. Treatment of SqTA in DMF with HMDS/MeOH at room temperature resulted in the overnight precipitation of SqTI-H, which was isolated in high purity at 71% yield by suction filtration. SqTI-H was found to be insoluble in most common organic solvents, with marginal room temperature solubility in DMF and DMSO (scarcely sufficient for ^1^H NMR analysis), that improves (to ∼1–2 mg mL^−1^) upon heating to temperatures approaching respective solvent boiling points. The poor solubility of SqTI-H is attributed to its hydrogen-bonding capabilities at the imide positions that strengthens solid state packing (see discussion of SqTI-H computations).

The chemical identity of SqTI-H was confirmed by its room temperature ^1^H NMR and 60 °C ^13^C{^1^H} NMR spectra (Fig. S9 and S10†). A broad peak at 3200 cm^−1^ was seen in the IR spectrum of SqTI-H and assigned to N–H stretching at the imide positions (Fig. S15†). N–H stretching was not observed in the IR spectra of SqTA and SqTI-iP (analysed henceforth to serve as a point-of-comparison representing alkyl-*N*-substituted SqTI-Rs), however, alkyl stretching peaks in the 2800–3000 cm^−1^ region were observed for SqTI-iP, assigned to its isopropyl sidechains. Strong C–N stretching originating at the imide positions were observed at ∼1340 cm^−1^ for both SqTI-H and SqTI-iP, which was not seen in the IR spectrum of SqTA, further proving successful conversion. As-synthesised SqTI-H is crystalline, as revealed by its powder X-ray diffraction (Fig. S16†). No peaks were observed in the differential scanning calorimetry traces of SqTI-H up to 300 °C (Fig. S17†), suggesting robust solid state packing and a melting point above 300 °C. The hydrogen-bonding capability of SqTI-H explains the presence of 3% w/w MeOH suggested by the elemental analysis of SqTI-H, despite overnight drying under high vacuum at 120 °C. MeOH retention in SqTI-H was further confirmed by its TGA trace (Fig. S14a†) that showed a gradual initial mass loss of 3% up to 250 °C, upon which mass loss behaviour stabilised until sample degradation at 550 °C. The pyrolysis temperature of SqTI-H is higher than that encountered for alkyl-*N*-substituted SqTI-Rs, indicating better thermal stability for SqTI-H.

**Fig. 1:**
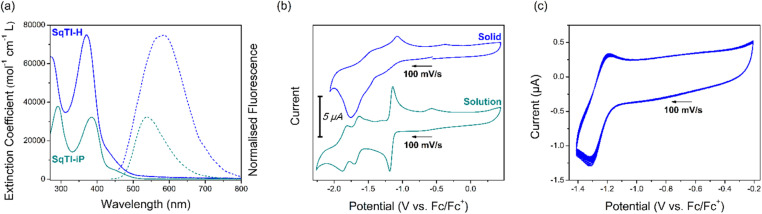
Optical and electronic properties of SqTI-H (blue) and SqTI-iP (dark cyan), showing (a) solution state UV/Vis (unbroken) and fluorescence (broken) traces of SqTI-H and SqTI-iP at ∼10 μM concentration in DMSO and CHCl_3_ respectively; (b) solid state CV of SqTI-H in 0.1 M [*n*-Bu_4_N]PF_6_/MeCN and solution state CV of SqTI-iP in 0.1 M [*n*-Bu_4_N]PF_6_/DMF; (c) 20 cycle solid state CV of SqTI-H with potential cut-off correspondent to accessing the globally aromatic dianion state. Arrows indicate scan direction.

Fig. 1a shows the solution state UV/Vis and fluorescence spectra of SqTI-H in DMSO and SqTI-iP in CHCl_3_. Two absorption bands within the optical windows of correspondent solvents were seen for both SqTI-H and SqTI-iP, at *λ*_max_ = 371/271 and 385/291 nm respectively. Fluorescence emission of SqTI-H with 371 nm excitation peaked at *λ*_max_ = 585 nm, whilst emission for SqTI-iP at 385 nm excitation peaked at *λ*_max_ = 539 nm. The large observed Stokes shifts of 1.22 and 0.92 eV for SqTI-H and SqTI-iP, respectively, are a signature of type II concealed antiaromaticity.^9,35^ By taking the intersection of absorption and normalised emission traces, the optical bandgaps of SqTI-H and SqTI-iP were estimated to be 2.7 and 2.6 eV respectively.

The solution state cyclic voltammetry of SqTI-iP was recorded in [*n*-Bu_4_N]PF_6_/DMF to reveal comparable electrochemical behaviour with previously reported alkyl-*N*-substituted SqTI-Rs (Fig. 1b, bottom).^16^ Cathodic scanning of SqTI-iP to −2.2 V *vs.* Fc/Fc^+^ uncovered three reversible electrochemical reduction processes, centred about −1.17, −1.67 and −1.84 V. Referencing the electrochemical behaviour of previous reported analogues,^16^ the process at −1.17 V can be assigned to a two-electron reduction to reach the globally aromatic dianion state of SqTI-iP that is confirmed by its forward/return peak-to-peak separation of 47 mV which is below the thermodynamic limit for a one-electron process (57 mV at 25 °C). SqTI-iP is subsequently reversibly reduced to the trianion and tetraanion states at −1.67 and −1.84 V respectively, which are one-electron processes according to correspondent peak-to-peak separations of 73 and 70 mV. The insolubility of SqTI-H limited its electrochemical study to the solid state, with CVs recorded in 0.1 M [*n*-Bu_4_N]PF_6_/MeCN using fine powder samples immobilised onto the working electrode surface as an electrolyte slurry. Cathodic scanning of SqTI-H to −1.4 V *vs.* Fc/Fc^+^ uncovered a reversible process centred about −1.25 V (Fig. 1c), assigned to reduction of SqTI-H to its globally aromatic dianion state, which was observed to be highly stable over 20 cycles despite data collection in the solid state. More SqTI-H electrochemical reduction processes were seen at applied potentials between −1.4 V to −2.0 V (Fig. 1b, top), at approximate potentials of −1.5 and −1.8 V respectively, which corresponds well with the potentials of the SqTI-iP reduction from the dianion > trianion > tetraanion. Solid state electrochemical reduction of SqTI-H from the dianion to the tetraanion was seen to be less reversible than that for alkyl-*N*-substituted SqTI-Rs in the solution state. From the electrochemical reduction potentials to each dianion, the electron affinities of SqTI-H and SqTI-iP were estimated to lie at −3.5 and −3.6 eV respectively (indicative of LUMO energy levels), from which deduction of optical bandgaps gives approximation of ionisation potentials (indicative of HOMO energy levels, Table 1). SqTI-iP and SqTI-H oxidations were not observed within the relevant electrochemical windows.

**Table 1:** Summary of SqTI-H and SqTI-iP electronic properties

Material	Reduction potential to dianion (V *vs*. Fc/Fc^+^)	Electron affinity (eV)	*E*_g_ (eV)	Ionisation potential (eV)
SqTI-H	−1.25	−3.5^a^	2.7^b^	−6.2^c^
SqTI-H (computed)	−1.23	−3.57^d^	2.58^e^	−6.15^f^
SqTI-iP	−1.17	−3.6^a^	2.6^b^	−6.2^c^

a Calculated from the reduction potential to dianion, by assuming the Fc/Fc^+^ process occurs at −4.8 eV.^36^

b From the intersection of UV/Vis and normalised fluorescence (to sample absorption at respective excitation wavelength) traces.

c Estimated by subtracting optical bandgap from the corresponding LUMO energy level.

d Determined as half the negative electron affinity for the dianion.

e By subtraction of computed ionisation potential from electron affinity.

f Determined as half the negative ionisation potential for the dication.

**Fig. 2:**
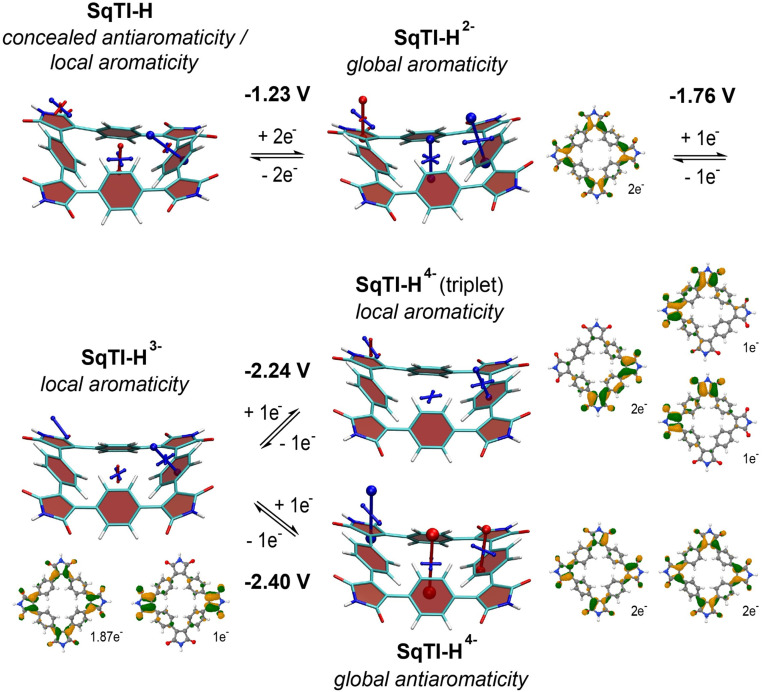
Computational analysis of SqTI-H: VIST plots in the neutral and reduced states showing the aromaticity switching upon reduction (shielded (aromatic) tensor components are shown in blue, deshielded (antiaromatic) tensor components in red), computed redox potentials for the different reduction steps in DMF, and dominant NDOs between the neutral state and the anions (green/orange NDOs for electron attachment).

To investigate whether the absence of alkyl sidechains in SqTI-H affects its ability to switch between different locally and globally aromatic states upon redox cycling, nucleus-independent chemical shift (NICS) calculations were performed for the different states.^37^ The NICS tensors are represented graphically using the visualisation of chemical shielding tensors (VIST) method^38^ showing them in the context of the molecular structure (Fig. 2). The shielding tensor components, displayed as blue (shielded, aromatic) or red (deshielded, antiaromatic) dumbbells in the VIST plots, relate to ring currents in the plane perpendicular to the respective dumbbell.

The VIST plots show that the antiaromaticity of the macrocyclic 4*n* π-electron system is effectively concealed in the neutral state (Fig. 2, left); the small red tensor component in the centre of neutral SqTI-H indicates at most weak antiaromatic/paratropic macrocyclic ring currents. The molecule is dominated by local aromaticity of the phenylene subunits, revealed by the large blue tensor component positioned at the phenylene pointing in a direction perpendicular to its plane, suggesting strong local aromatic/diatropic ring currents. No noticeable aromaticity was seen for the imide ring. However, as also reported for PCT and alkyl-*N*-substituted SqTI-Rs,^8,16^ SqTI-H transitions to a globally aromatic state upon its two-electron reduction, as evidenced by the enhanced shielding inside the ring (−22.5 ppm) and the alignment of the phenylene VIST tensor with the macrocyclic ring current. The natural difference orbital (NDO) on reduction to the dianion is provided below the VIST plot, showing spatial occupancy of the two electrons upon charging onto SqTI-H. The NDO of the SqTI-H dianion shows enhanced contributions on the electron-deficient imide rings with almost no contributions on the phenylene units, which is different to PCT (and its substituted analogues without imide groups) with more evenly delocalised dianion NDOs,^19,35,39^ highlighting the strong influence of the imide groups on the redox properties of SqTI-H. Computations show further reduction of SqTI-H from the dianion results in a locally aromatic trianion, followed by a locally aromatic triplet tetraanion, that is lower in energy than the globally antiaromatic singlet tetraanion, which shows a strongly deshielded VIST tensor component at the centre (+51.8 ppm). The computed redox potentials for reduction of SqTI-H to the triplet and singlet tetraanions are −2.24 V and −2.40 V, respectively. The indicated local aromaticity of the SqTI-H triplet tetraanion herein contrasts against the global Baird aromaticity of the triplet tetraanions of alkyl-*N*-substituted SqTI-Rs from our previous study.^16^

**Fig. 3:**
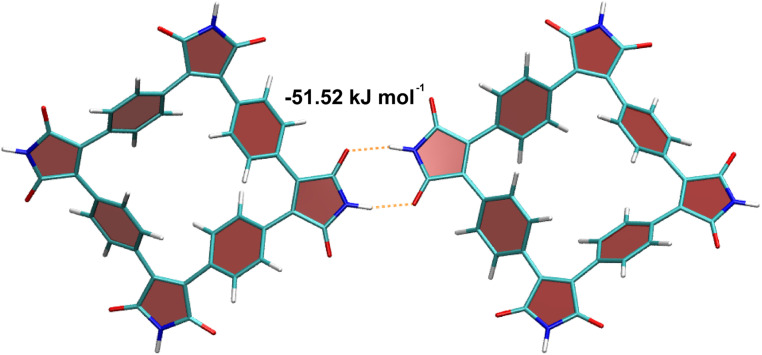
Computed structure of the SqTI-H dimer, showing its capability to form intermolecular double hydrogen-bonding interactions. Atom colours are: carbon – dark cyan, oxygen – red, nitrogen – blue and hydrogen – white.

The unique feature of SqTI-H compared to alkyl-*N*-substituted SqTI-Rs is its potential to form hydrogen-bonding interactions, which was investigated using density functional theory (DFT) to see whether this explains its low solubility. A SqTI-H dimer structure featuring two intermolecular hydrogen-bonding interactions between adjacent imide groups was localised by DFT (Fig. 3), with each imide moiety engaging in one donor and one acceptor hydrogen-bonding interaction, reminiscent of nucleobase pairing or more specifically, the pyridone dimer.^40,41^ The double hydrogen-bonding interaction emanating from each imide on the SqTI-H macrocycle is associated with a substantial interaction energy of 51.5 kJ mol^−1^, which is just less than half the computed dimer stacking energy (123.5 kJ mol^−1^), suggesting hydrogen-bonding is a strong contributor to the overall interaction energy within the SqTI-H solid.

**Fig. 4:**
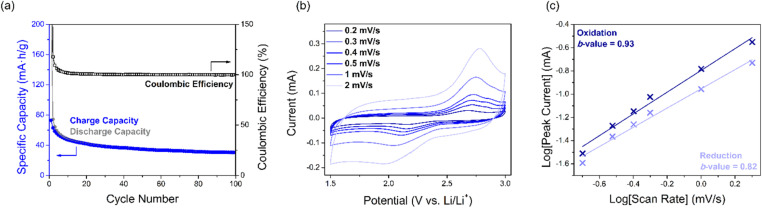
Performance of SqTI-H composite electrodes in 1 M LiTFSI/1 : 1 DOL + DME, showing (a) cycling performance and coulombic efficiency, (b) scan rate dependence CV, as well as (c) log[scan rate]–log[peak current] plot of data from (b) to extract *b*-values.

To evaluate the energy storage performance of SqTI-H, composite electrodes (SqTI-H/Ketjen Black/PVDF: 50/40/10) were fabricated and investigated in lithium–organic half cells. Ketjen Black was selected as the conductive carbon additive owing to its microporous structure,^42^ which could aid SqTI-H active material immobilisation. SqTI-H electrodes were first evaluated by CV (Fig. S18a†) in 1 M LiTFSI/1 : 1 1,3-dioxolane (DOL) and 1,2-dimethoxyethane (DME), where in the first cycle two reduction peaks at 2.2 and 1.9 V *vs.* Li/Li^+^ as well as one oxidation peak at 2.6 V were observed. The peak at 2.2 V can be assigned to SqTI-H reduction to the globally aromatic dianion, the intensity of which decreases upon cycling, indicating dissolution of the reduced species in the battery electrolyte, whilst the peak at 1.9 V completely disappears in subsequent cycles, suggesting its tentative assignment to NH lithiation. During constant current cycling at 50 mA g^−1^ (0.5C), SqTI-H shows an initial discharge capacity of 226 mA h g^−1^, which exceeds its theoretical specific capacity (156 mA h g^−1^, assuming 4-electron transfer), and an initial charge capacity of 73 mA h g^−1^ (Fig. 4a and S18b†). This irreversible capacity observed for the first discharge cycle is attributed to lithiation of NH groups. In the second cycle, SqTI-H attains a specific capacity of 75 mA h g^−1^ with high coulombic efficiency, but the discharge and charge capacities further diminish upon subsequent cycling, which is most likely caused by slow dissolution of macrocycle into the electrolyte. After 100 cycles, the SqTI-H electrode reaches a capacity approaching that of pure conductive carbon additive Ketjen Black (see Fig. S19† for the capacity contribution of Ketjen Black).

The electrochemical kinetics of the SqTI-H composite electrode was investigated using scan rate dependence CV (Fig. 4b), the data for which were fit according to the power law equation, *i*_p_ = *aν*^*b*^, where *i*_p_ is the peak current, *ν* is the scan rate, and *a*/*b* are adjustable parameters. The fitted *b*-value indicates whether electrochemical reduction of the SqTI-H composite electrode is diffusion controlled (*b*-value close to 0.5) or capacitive (*b*-value close to 1).^43^ By plotting log[*ν*] against log[*i*_p_] of data from Fig. 4b for both reduction and oxidation peak currents, the cathodic and anodic *b*-values were determined as the correspondent slopes of linear regression to be 0.82 and 0.93 respectively, indicating more capacitive electrochemical behaviour for the SqTI-H composite electrode.

To further improve the energy storage performance of SqTI, its reduced state solubility in the battery electrolyte should be minimised, for example by attaching ionic groups, or by pre-lithiation/metallation at the nitrogen position, together with the use of a mesoporous conductive carbon additive, which can better confine the active material during cycling.

## Conclusions

Despite the favourable redox properties of alkyl-*N*-substituted SqTI-Rs, their excellent solubilities prevent their stable application as battery electrode materials for organic energy storage. In response, we developed unsubstituted SqTI-H, which was synthesised in good yield by the facile, one-step conversion of SqTA with HMDS and MeOH. Physical characterisation of as-synthesised SqTI-H indicated sidechain removal to be an effective strategy in not only reducing compound solubility, but also improving thermal stability, in comparison with alkyl-*N*-substituted SqTI-Rs. The enhancement in physical properties was explained by the computationally demonstrated hydrogen-bonding capabilities emanating from each unsubstituted imide group on SqTI-H. Reversible electrochemical reduction of SqTI-H in the solid state to reach the globally aromatic dianion was also observed at −1.25 V *vs.* Fc/Fc^+^, along with two further reduction steps. The physical robustness and redox-active nature of SqTI-H suggests potential good performance as a battery electrode material. However, preliminary testing in lithium–organic half cells showed unstable cycling performance, which may be explained by solubility of the lithiated, reduced species. Further optimisations by SqTI-H pre-treatment and of electrode composition are underway, with the aim of minimising the solvation of reduced SqTI-H. This study demonstrates the success of sidechain removal as a strategy in reducing neutral state solubility of SqTI. However, in terms of device performance, our results highlight the importance of considering the potential lithiation of unsubstituted imides at the nitrogen position as well as the reduced state solubilities in the design of conjugated macrocycles for energy storage.

## Experimental methods

### Materials

All reagents and solvents were obtained commercially and used without further purification unless otherwise stated. SqTA was synthesised according to literature procedure, the characterisation of which matched that reported.^16^

### Synthesis

#### SqTI-iP

SqTA (0.20 g, 0.29 mmol) was suspended in THF (5 mL) and the solution was deoxygenated with N_2_ for 15 min. Isopropylamine (0.50 mL, 5.8 mmol) was added, and the solution was stirred at room temperature for 2 h, followed by overnight heating at 100 °C in a sealed vial. The solution was allowed to cool to room temperature, and the volatiles were removed *in vacuo* to give the desired product as a bright orange solid (0.20 g, 81%). ^1^H NMR (CDCl_3_, 400 MHz, 298 K): *δ* 7.70 (s, 16H, Ar–H), 4.47 (quint, ^3^*J*_H–H_ = 6.8 Hz, 4H, N–**CH**–(CH_3_)_2_), 1.46 (d, ^3^*J*_H–H_ = 6.8 Hz, 24H, –CH_3_) ppm. ^13^C{^1^H} NMR (CDCl_3_, 100 MHz, 298 K) *δ* 169.9, 134.9, 130.4, 130.3, 43.7, 20.2 ppm. HRMS (APCI^−^): calculated for C_52_H_44_N_4_O_8_ [M]^−^, 852.3165; found, 852.3145.

#### SqTI-Hp

SqTA (0.050 g, 0.073 mmol) and 4-heptylamine (0.16 g, 0.20 mL, 1.40 mmol) were dissolved in THF, and deoxygenated with N_2_ for 15 min. The solution was stirred at room temperature for 2 h, followed by reflux overnight. Upon cooling, the volatiles were removed *in vacuo* to give the product as a bright orange solid (0.052 g, 66%). ^1^H NMR (CDCl_3_, 400 MHz, 298 K): *δ* 7.72 (s, 16H, Ar–H), 4.17 (m, 4H, N–**CH**–(CH_2_)_2_), 2.01 (m, 8H, CH–**CH_2_**–CH_2_), 1.64 (m, 8H, CH–**CH_2_**–CH_2_), 1.28 (m, 16H, CH_2_–**CH_2_**–CH_3_), 0.91 (t, ^3^*J*_H–H_ = 7.2 Hz, 24H, –CH_3_) ppm. ^13^C{^1^H} NMR (CDCl_3_, 100 MHz, 298 K) *δ* 170.3, 134.5, 130.5, 130.4, 52.4, 34.6, 20.0, 13.9 ppm. HRMS (APCI^−^): calculated for C_68_H_76_N_4_O_8_ [M]^−^, 1076.5669; found, 1076.5673.

#### SqTI-tBu

SqTA (0.050 g, 0.073 mmol) and *tert*-butylamine (0.10 g, 0.16 mL, 1.40 mmol) were dissolved in THF, and deoxygenated with N_2_ for 15 min. The solution was stirred at room temperature for 2 h, followed by reflux overnight. Upon cooling, the volatiles were removed *in vacuo* to give the product as a bright orange solid (0.036 g, 54%). ^1^H NMR (CDCl_3_, 400 MHz, 298 K): *δ* 7.59 (s, 16H, Ar–H), 1.66 (s, 36H, –CH_3_) ppm. ^1^H NMR (DMSO-*d*_6_, 400 MHz, 298 K): *δ* 7.48 (s, 16H, Ar–H), 1.61 (s, 36H, –CH_3_) ppm. ^13^C{^1^H} NMR (CDCl_3_, 100 MHz, 298 K) *δ* 171.1, 135.0, 130.4, 130.2, 58.2, 29.1 ppm. HRMS (APCI^−^): calculated for C_56_H_52_N_4_O_8_ [M]^−^, 908.3791; found, 908.3783.

##### Attempted acidic cleavage of SqTI-tBu

SqTI-tBu (0.010 g, 0.011 mmol) was added to trifluoroacetic acid (10 mL) and refluxed in air for 16 h. The resulting mixture was dried *in vacuo* to give the crude product as a dull orange solid that was analysed by ^1^H NMR without further purification (see Fig. S8†).

#### SqTI-H

SqTA (0.14 g, 0.20 mmol) was dissolved in DMF (2 mL) in air, to which hexamethyldisilazane (0.93 mL, 1.3 g, 8.1 mmol) and MeOH (0.16 mL, 0.13 g, 4.1 mmol) were added in that order, resulting in the formation of a cream precipitate that darkened to an orange solid upon overnight stirring at RT. The orange solid was isolated by filtration, washing with MeOH, to give SqTI-H (0.098 g, 71%, mp ≥ 300 °C). ^1^H NMR (DMSO-*d*_6_, 400 MHz, 298 K): *δ* 11.37 (s, 4H, N–H), 7.53 (s, 16H, Ar–H) ppm. ^13^C{^1^H} NMR (DMSO-*d*_6_, 100 MHz, 333 K): *δ* 170.9, 135.4, 129.7, 129.5 ppm. Elemental analysis: found, %: C 68.69, H 2.74, N 7.53. Calculated for (C_40_H_20_N_4_O_8_)_3_·(CH_4_O)_2_, %: C 69.18, H 3.24, N 7.93.

### Computational details

Geometries of SqTI-H in its various charged and spin states were optimised in vacuum with density functional theory (DFT) using the PBE0 functional^44,45^ along with the def2-SV(P) basis set^46^ and the D3 dispersion correction.^47^ Vibrational analyses were performed at the same level of theory and thermostatistical corrections were computed using the quasi-rigid-rotor-harmonic-oscillator model.^48^ Additional single-point computations were performed at the PBE0/def2-SVPD level of theory using the SMD solvent model to represent dimethylformamide.^49^ The reported electron affinities and ionisation potentials were computed by combining the solvated PBE0/def2-SVPD single point energies with gas-phase PBE0/def2-SV(P) thermostatistical corrections. Redox potentials were computed as previously described^35^ using a value of 4.80 V for the reference electrode. These computations were carried out using Q-Chem 6.1.^50^ The hydrogen-bonded and stacked dimers were optimised at the r^2^SCAN-3c level^51^ as implemented in Orca 5.0.^52^

Chemical shielding tensors were computed at the PBE0/def2-SVP level using gauge including atomic orbitals^53^ as implemented in Gaussian 09.^54^ Shielding tensors were represented graphically using the VIST (visualisation of chemical shielding tensors) method^38^ as implemented in TheoDORE 3.0.^55^

### Lithium-ion battery electrode fabrication and characterisation

Electrodes were prepared by mixing 50 wt% of SqTI-H with 40 wt% of Ketjen Black (EC-600JD) and 10 wt% of polyvinylidene fluoride (PVDF, Solef 5130, BASF) using dry *N*-methyl-2-pyrrolidone (NMP) as solvent. The Ketjen Black electrodes were prepared by mixing 40 wt% of Ketjen Black and 60 wt% of PVDF binder in dry NMP. The slurries were coated on Cu foil using a doctor blade, and electrodes were dried overnight under vacuum at 80 °C. Coin cells (type 2032) were assembled in an argon-filled glove box with water and oxygen levels under 0.5 ppm. Lithium (0.75 mm thick, 12 mm diameter, Gelon lib group) was used as the counter electrode, a glass fibre membrane (Whatman, GF/D) was used as the separator and 1 M LiTFSI/1 : 1 DOL + DME was used as electrolyte. CV and galvanostatic experiments were performed with a BioLogic MPG 2 potentiostat.

## Conflicts of interest

There are no conflicts to declare.

## Supplementary Material

FD-250-D3FD00145H-s001
