# Supplementary material for: Reducing undesired solubility of squarephaneic tetraimide for use as an organic battery electrode material
Source: Faraday Discuss. 2023 Aug 17;250:129–44. doi: 10.1039/d3fd00145h (PMC10926975; doi:10.1039/d3fd00145h)
Supplement: FD-250-D3FD00145H-s001 [file FD-250-D3FD00145H-s001.pdf]

Electronic Supplementary Material (ESI) for Faraday Discussions.
This journal is © The Royal Society of Chemistry 2023

Supplementary Information

Reducing Undesired Solubility of Squarephaneic Tetraimide for Use as an Organic Battery Electrode Material

Bowen Ding,[a] Manik Bhosale,[b] Troy L.R. Bennett,[a] Martin Heeney,[a,c] Felix Plasser,[d] Birgit Esser,[b]* and Florian Glöcklhofer[a,e]*

[a]Department of Chemistry and Centre for Processable Electronics, Imperial College London, Molecular Sciences Research Hub (White City Campus), 80 Wood Lane Shepherd's Bush, London W12 0BZ, United Kingdom

[b]Institute of Organic Chemistry II and Advanced Materials, Ulm University, Albert-Einstein-Allee 11, 89081 Ulm, Germany

[c]Physical Sciences and Engineering Division (PSE), King Abdullah University of Science and Technology (KAUST), Thuwal 23955-6900, Saudi Arabia

[d]Department of Chemistry, Loughborough University, Loughborough LE11 3TU, United Kingdom

[e]Institute of Applied Synthetic Chemistry, TU Wien, Getreidemarkt 9/163, 1060 Vienna, Austria

*Corresponding Authors: Birgit Esser (birgit.esser@uni-ulm.de) & Florian Glöcklhofer (f.glocklhofer@imperial.ac.uk, florian.gloecklhofer@tuwien.ac.at)

GENERAL METHODS

UV/Vis data were collected using an Agilent CARY 60 UV/Vis spectrometer, whilst an Agilent Eclipse Fluorescence spectrophotometer was used for the collection of fluorescence data (both interfaced with SCAN software), for samples dissolved in either spectroscopic grade $CHCl_3$ or DMSO. Elemental analyses were performed through the Elemental Analysis Service Team at London Metropolitan University (United Kingdom), the samples for which were prepared by overnight drying under high vacuum at 120 °C.

ELECTROCHEMISTRY

Solution and solid state cyclic voltammetry (CV) measurements were conducted with a Metrohm Autolab PGSTAT101 Electrochemical Analyser interfaced to NOVA software. A one compartment three electrode electrochemical cell was used for all measurements, featuring a 7.1 mm^2 glassy carbon working electrode (WE) and a Pt counter electrode. Solid and solution state measurements were conducted in 0.1 M [n-Bu_4N]PF_6/MeCN and [n-Bu_4N]PF_6/DMF respectively, with use of an Ag/Ag^+ non-aqueous reference electrode and addition of ferrocene

internal reference. Saturation of the electrolyte with N_2 by bubbling for 20 mins was performed to deoxygenate before measurements were taken. Solid state samples were immobilised onto the WE surface by dipping the electrode into a paste made with MeCN. [n-Bu_4N]PF_6 for electrochemistry was recrystallized twice in EtOH.

NUCLEAR MAGNETIC RESONANCE (NMR) SPECTROSCOPY

Solution state ^1H and ^{13}C NMR spectra were collected on either a Bruker AVANCE 400 or 500 MHz spectrometer. Deuterated $CDCl_3$ and DMSO-d_6 for NMR were obtained from Sigma Aldrich, and their solvent residual signals were used as internal references for chemical shifts (δ).

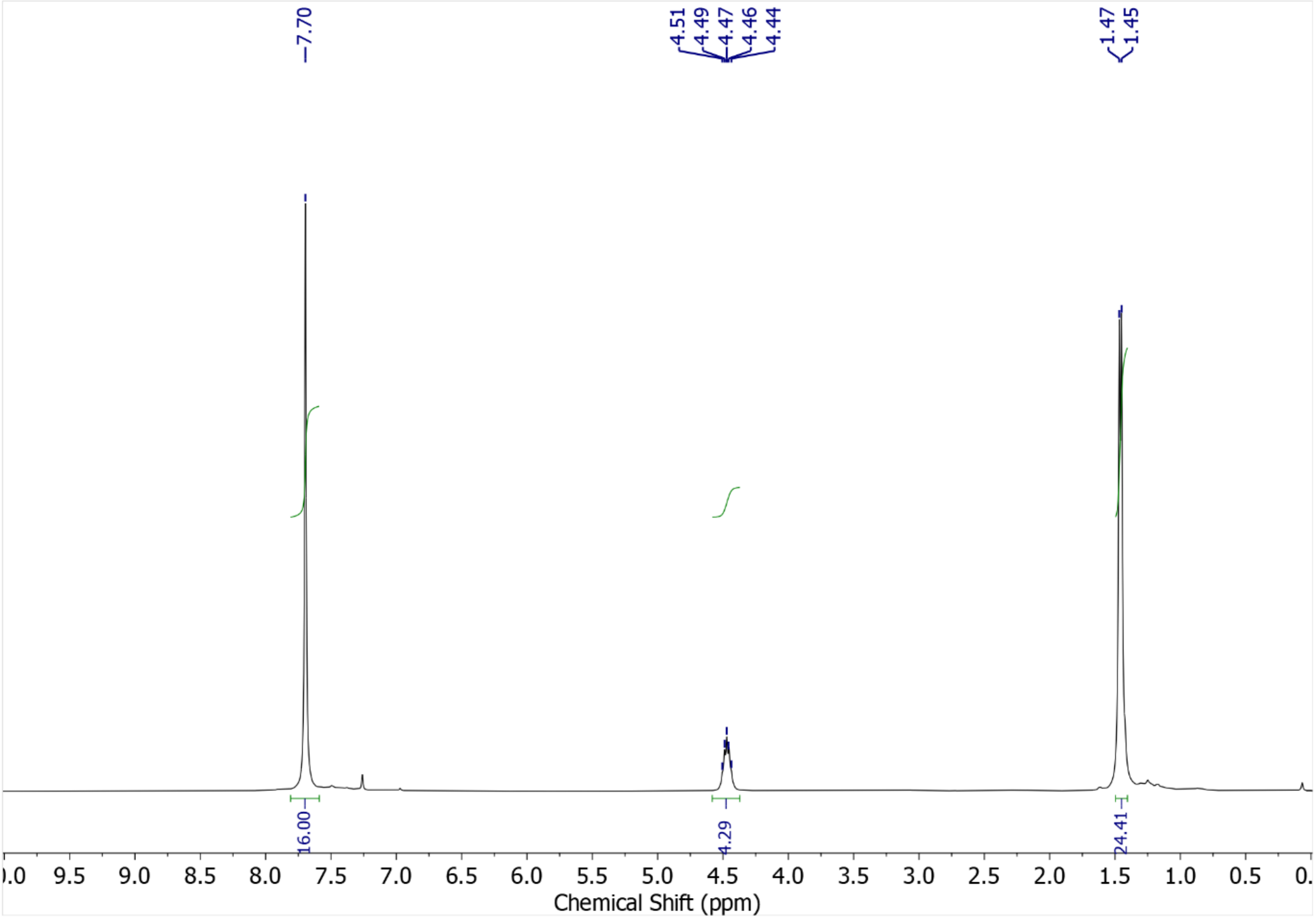

Figure S1: ^1H NMR of SqTI-iP in $CDCl_3$ at 298 K.

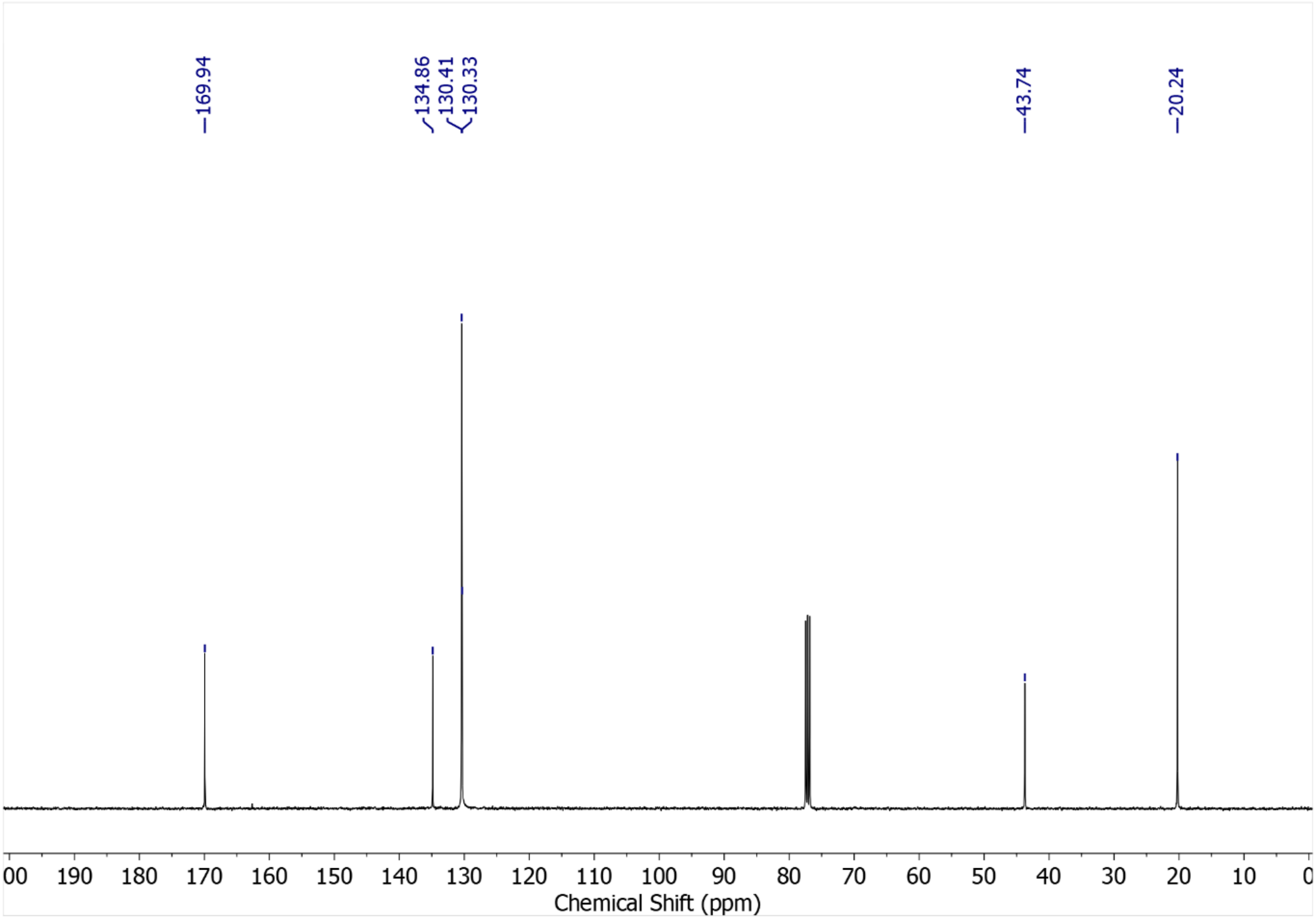

Figure S2: $^{13}C\{^1H\}$ NMR of SqTI-iP in $CDCl_3$ at 298 K.

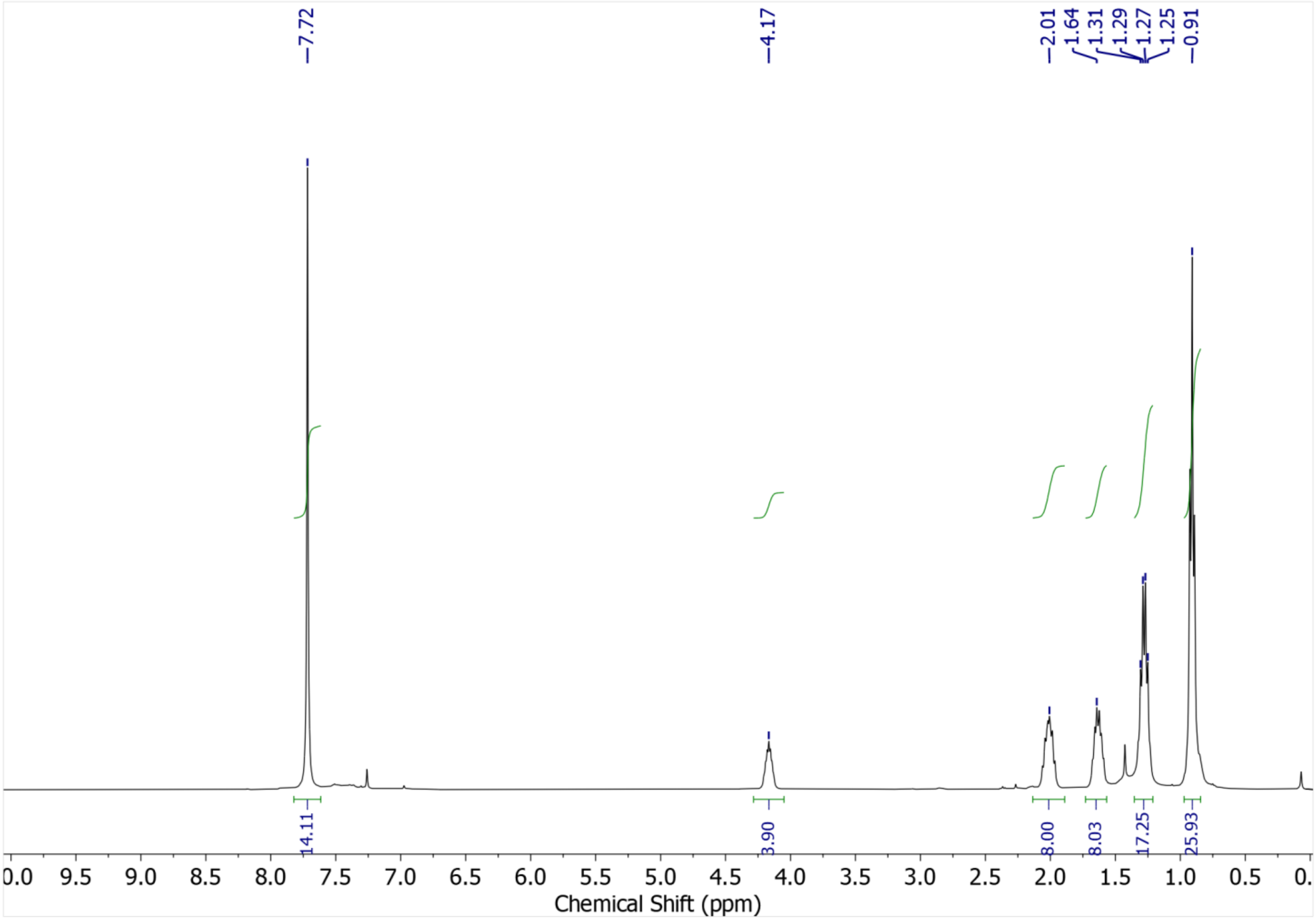

Figure S3: ^1H NMR of SqTI-Hp in $CDCl_3$ at 298 K.

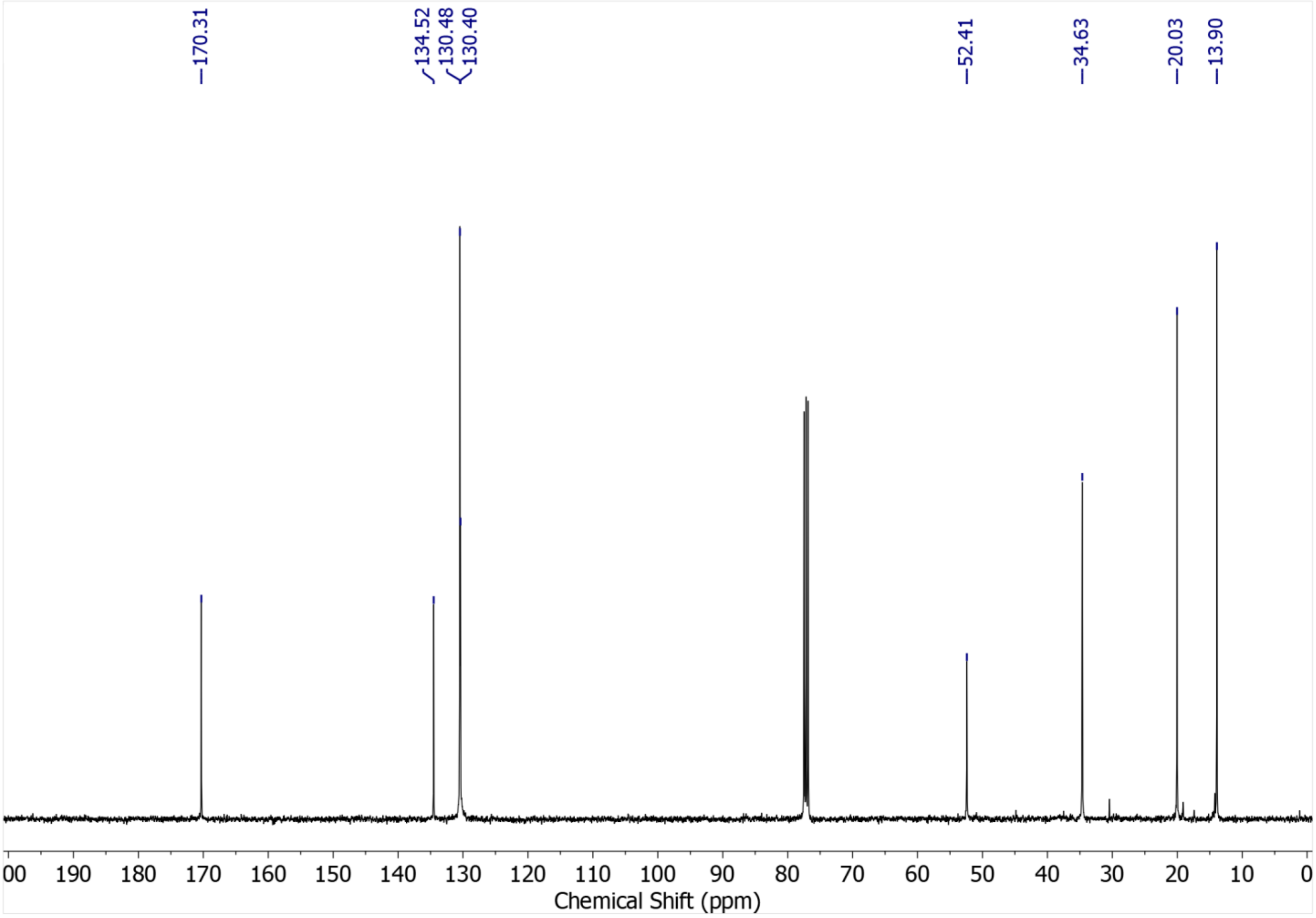

Figure S4: $^{13}C\{^{1}H\}$ NMR of SqTI-Hp in $CDCl_3$ at 298 K.

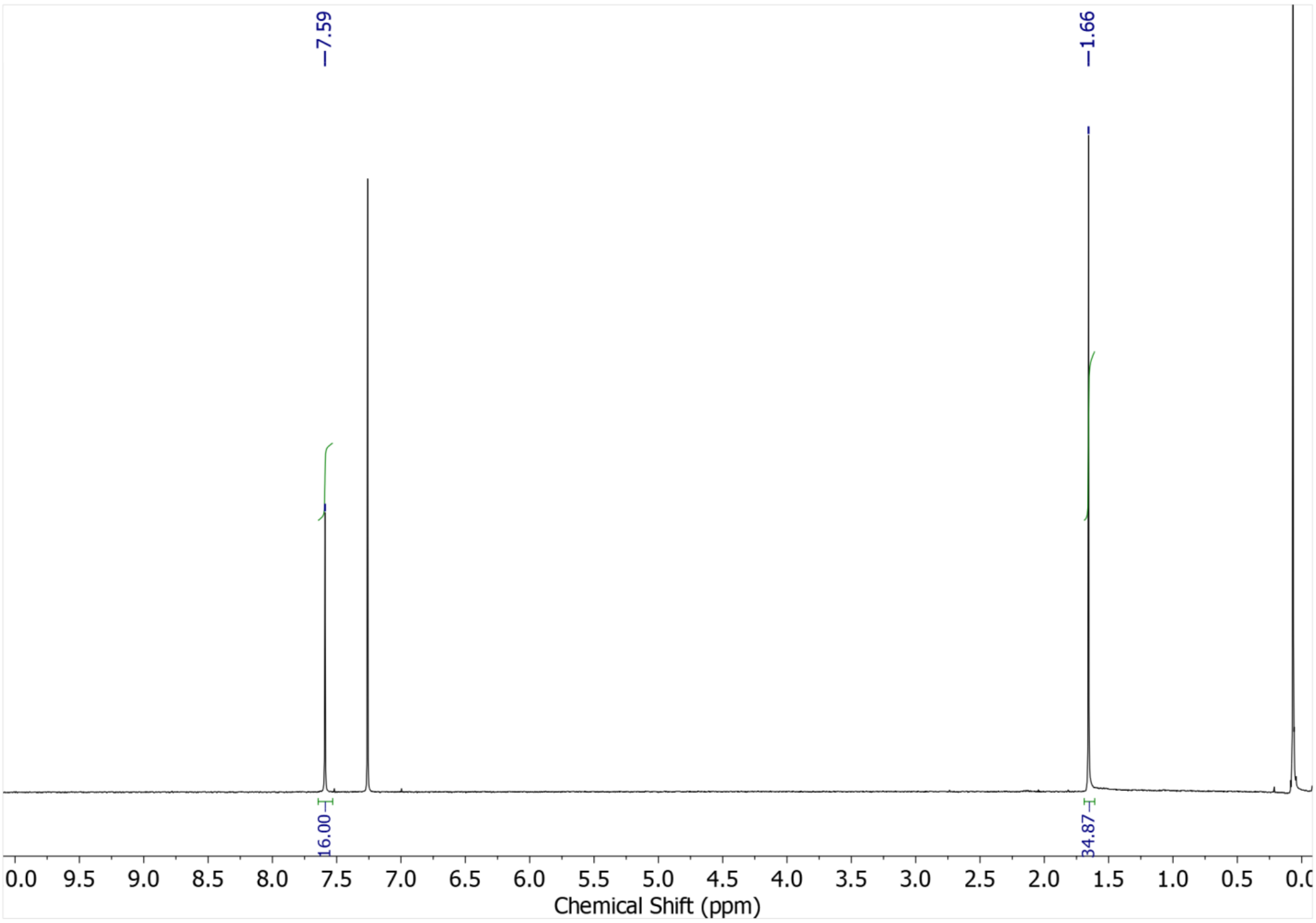

Figure S5: ^{1}H NMR of SqTI-tBu in $CDCl_3$ at 298 K.

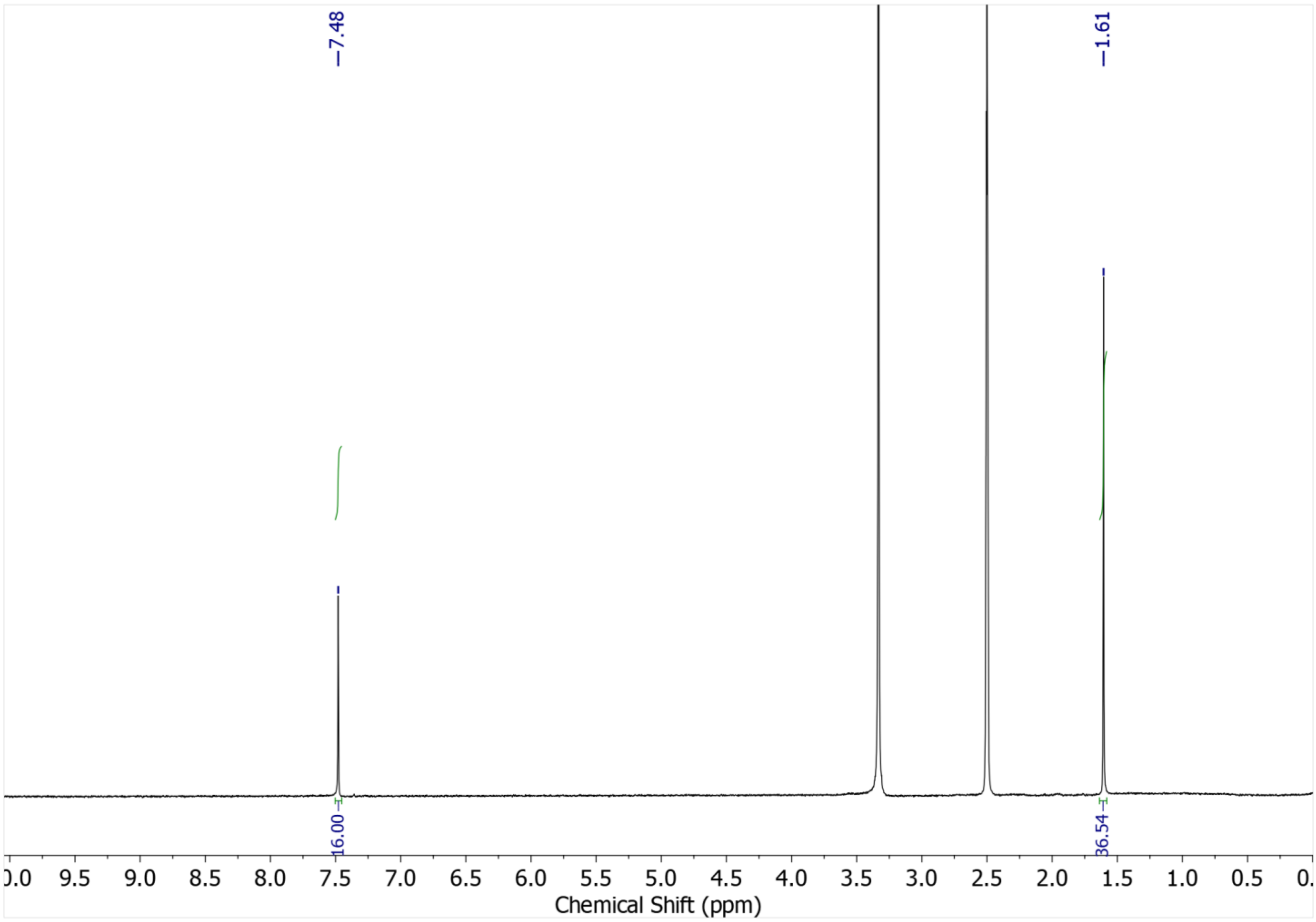

Figure S6: ^{1}H NMR of SqTI-tBu in DMSO-d_6 at 298 K.

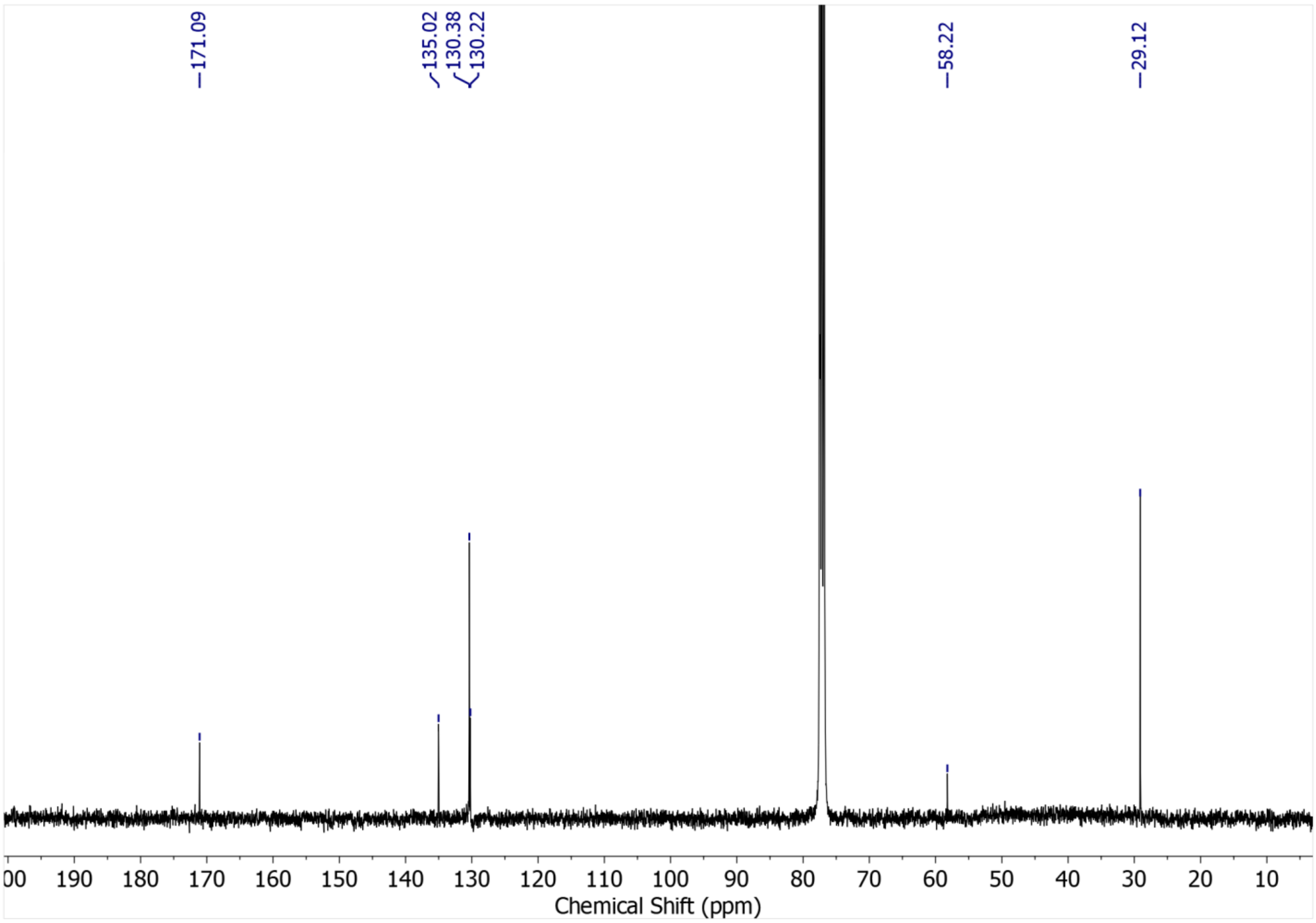

Figure S7: $^{13}C\{^{1}H\}$ NMR of SqTI-tBu in $CDCl_3$ at 298 K.

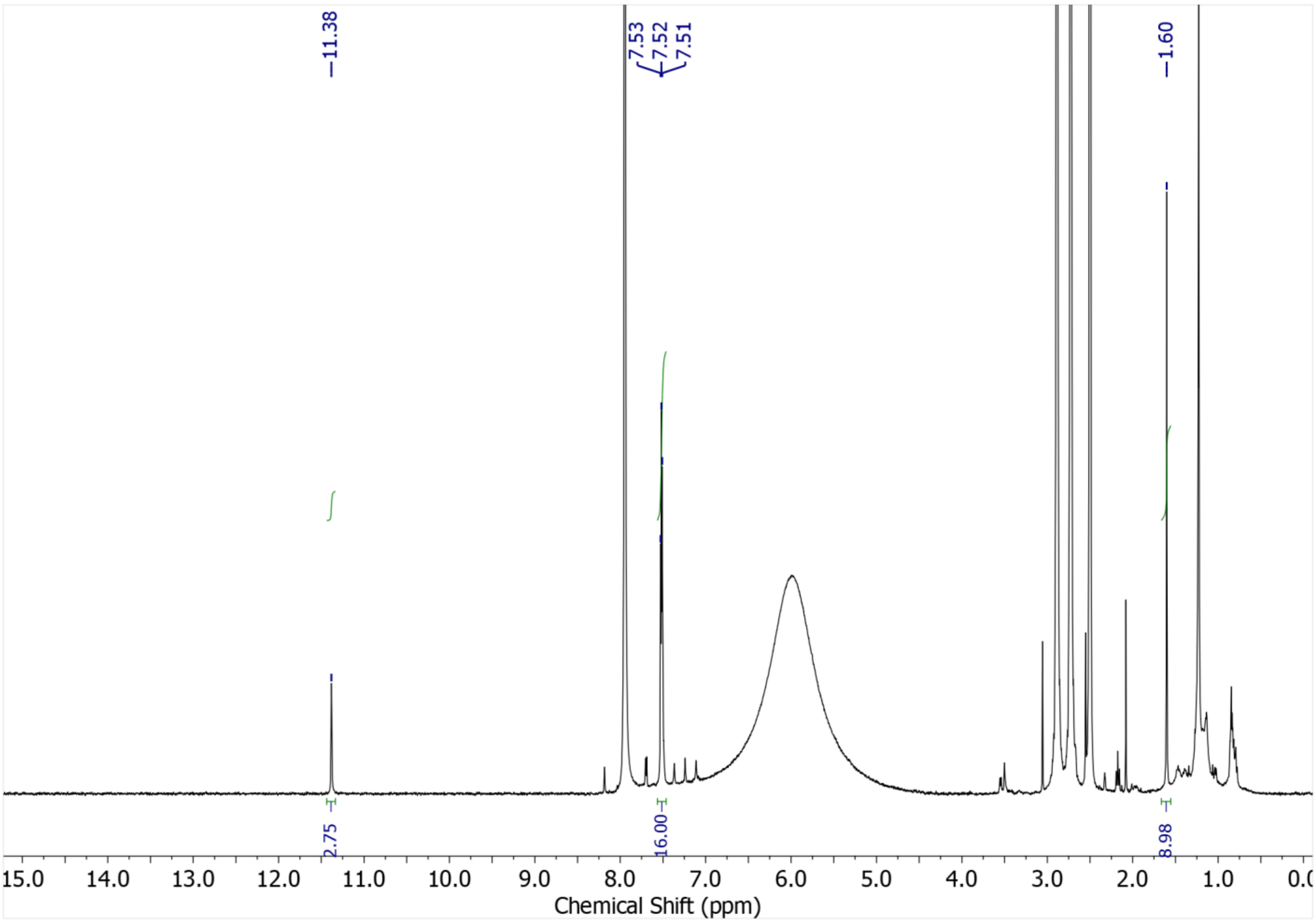

Figure S8: ^{1}H NMR of SqTI-tBu after attempted acidic sidechain cleavage by overnight refluxing in trifluoracetic acid, in DMSO-d_6 at 298 K.

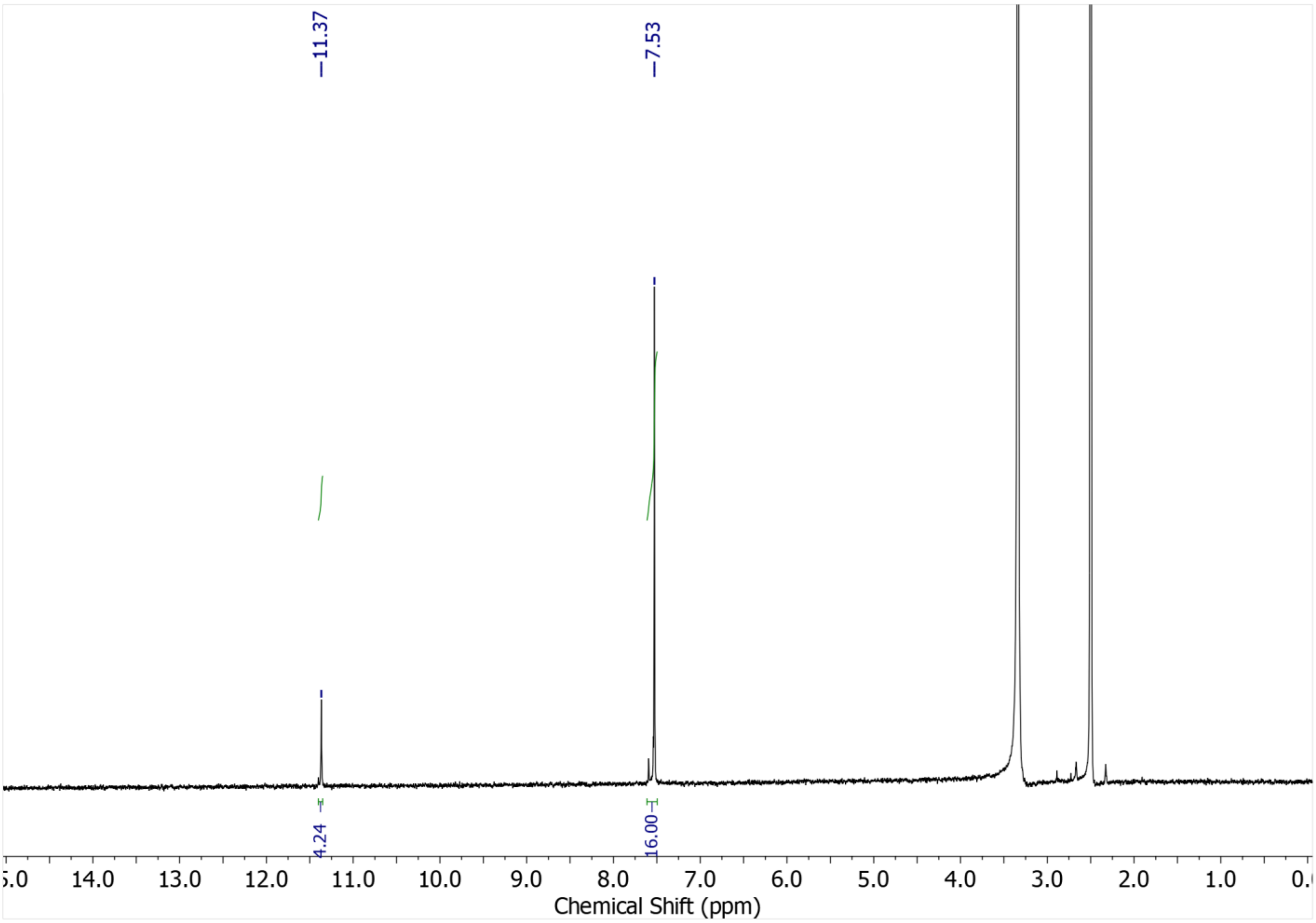

Figure S9: ^{1}H NMR of SqTI-H in DMSO-d_6 at 298 K.

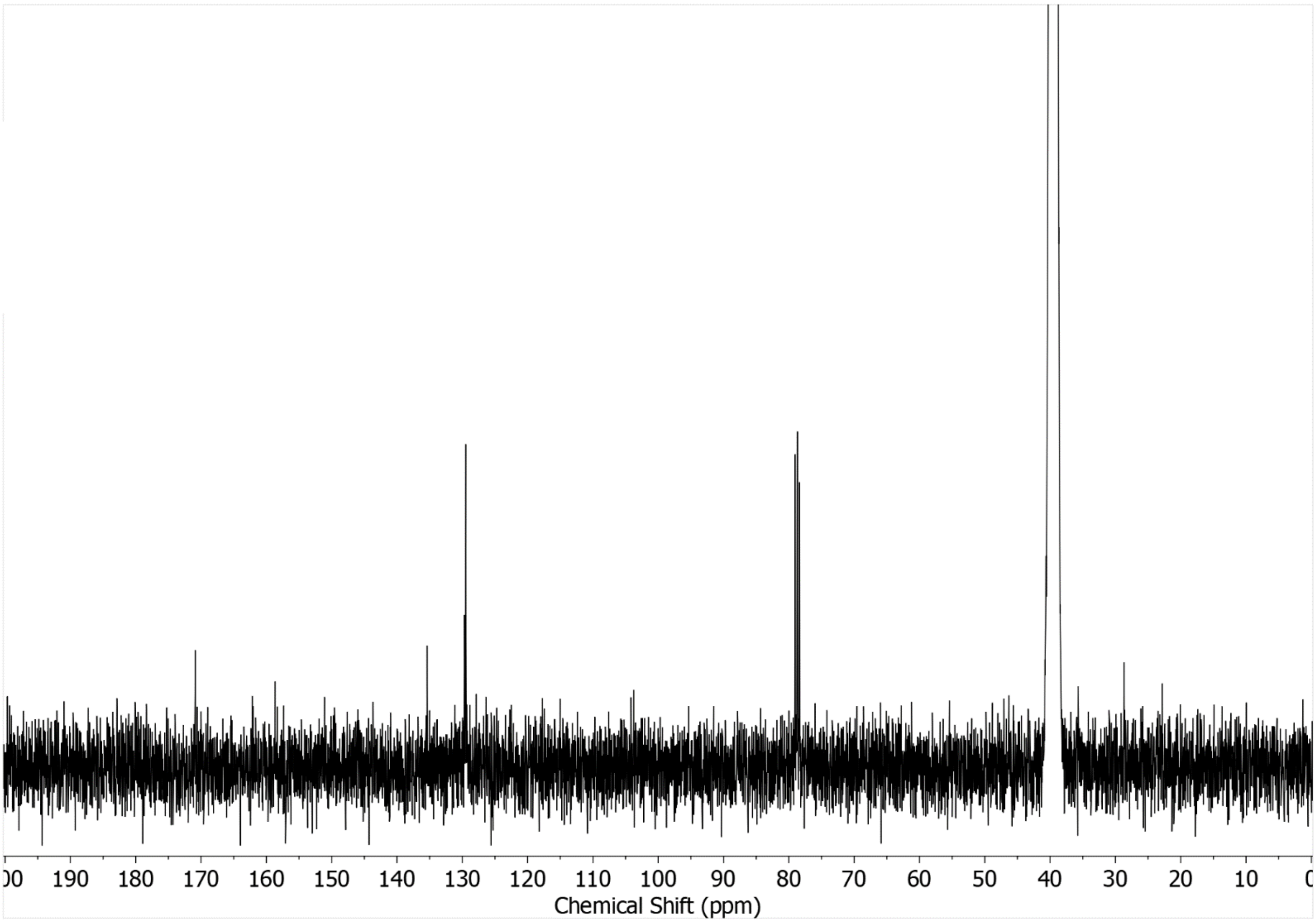

Figure S10: $^{13}C\{^{1}H\}$ NMR SqTI-H in DMSO-d_6 at 333 K.

HIGH-RESOLUTION MASS SPECTROMETRY (HRMS)

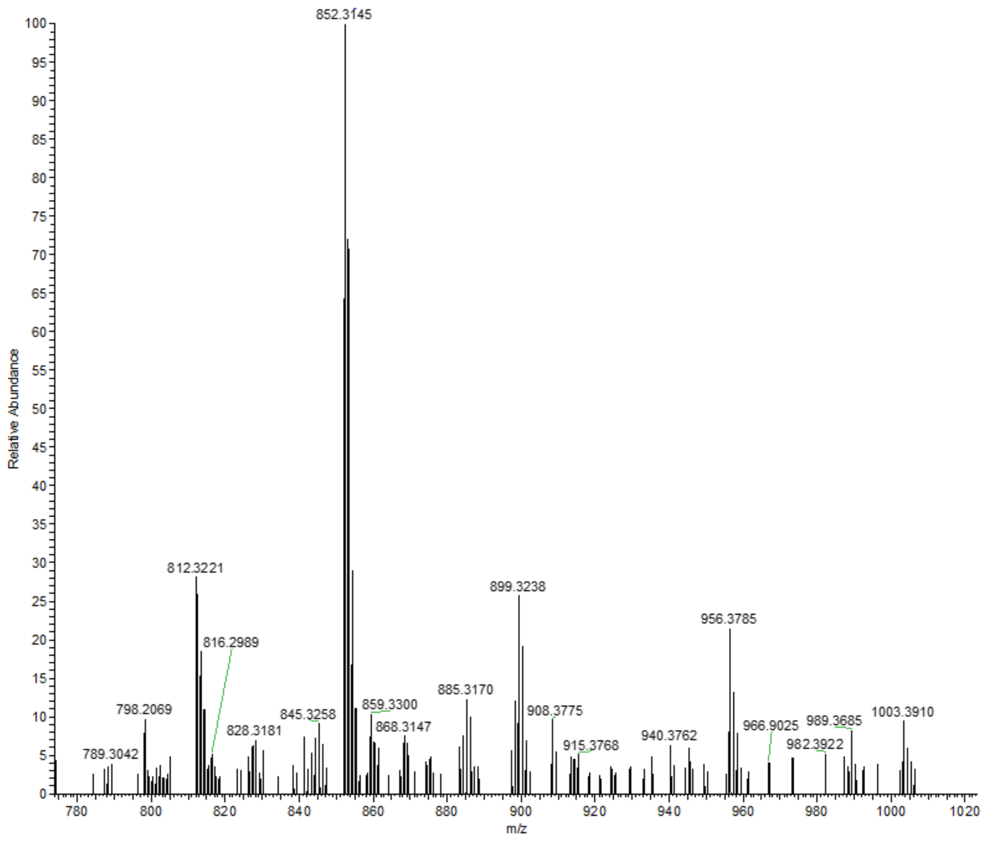

Figure S11: HRMS trace of SqTI-iP.

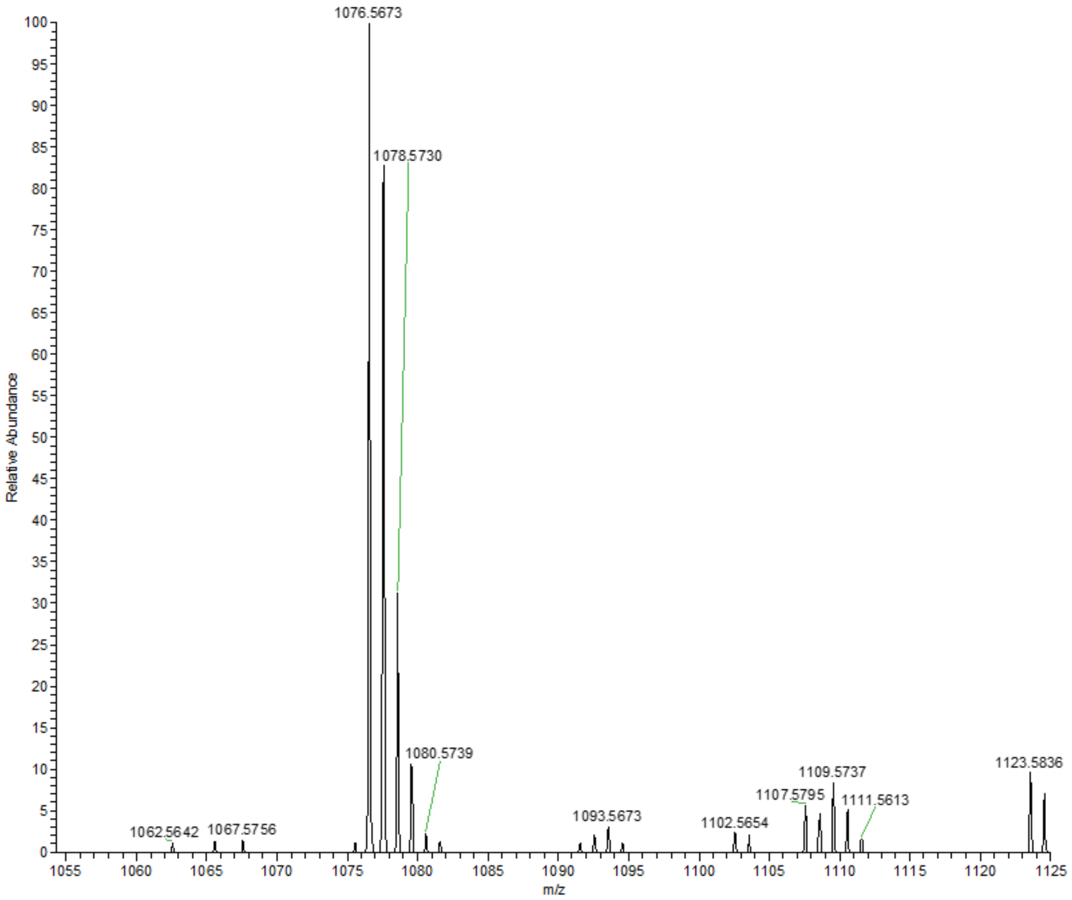

Figure S12: HRMS trace of SqTI-Hp.

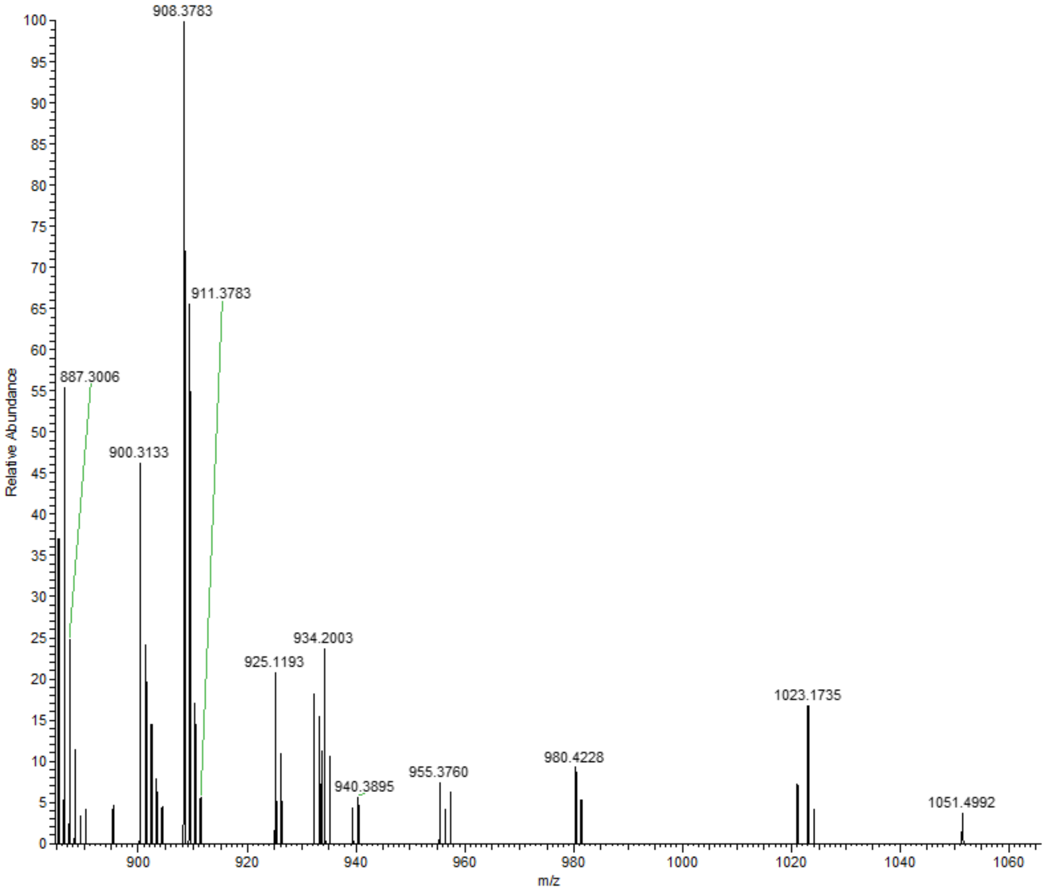

Figure S13: HRMS trace of SqTI-tBu.

THERMOGRAVIMETRIC ANALYSIS (TGA)

Thermogravimetric analyses (TGA) were performed on a Mettler Toledo TGA at a heating rate of 10 °C min^{-1}, under a constant stream of N_2 at a flow rate of *ca*. 50 mL min^{-1}.

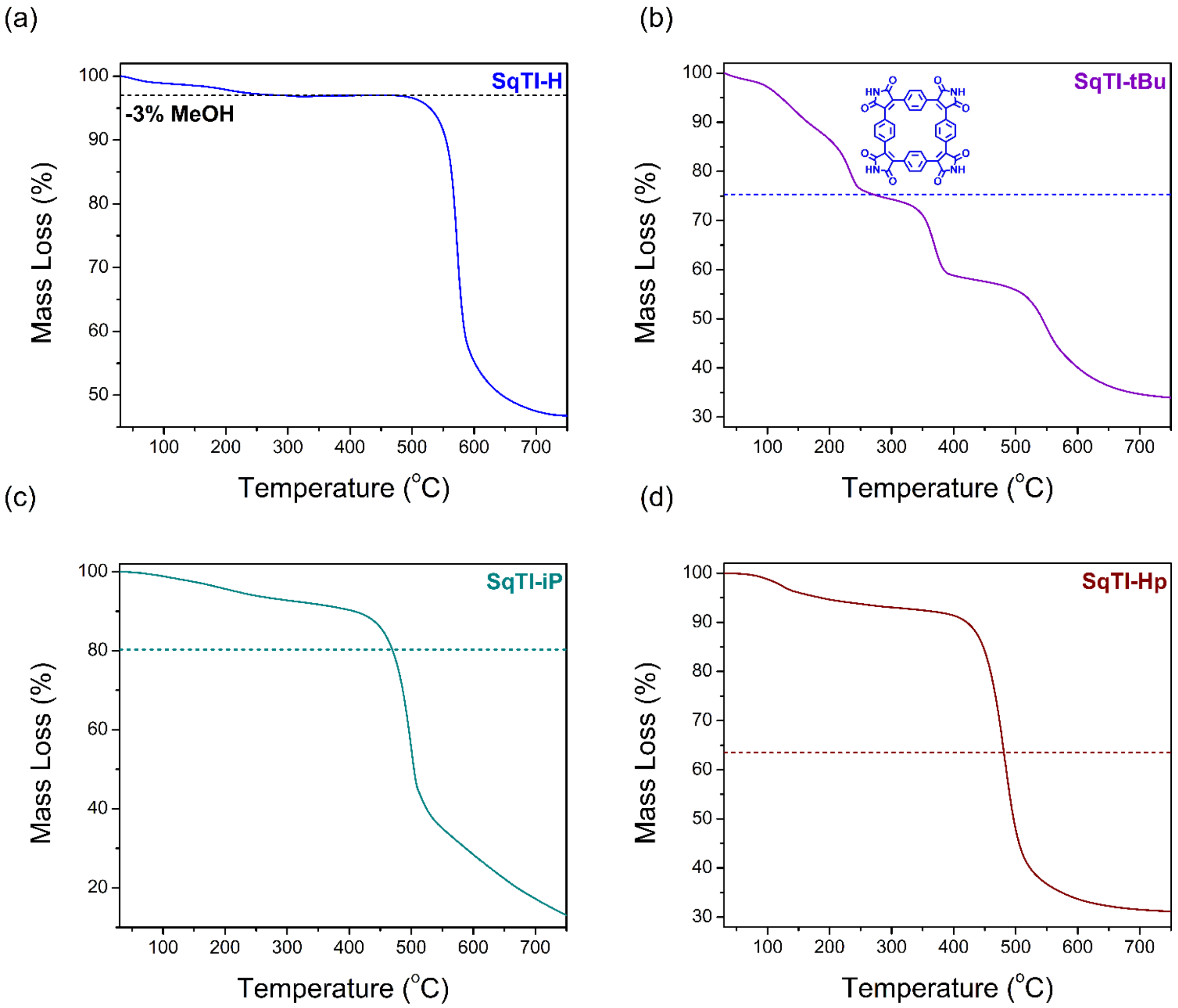

Figure S14: TGA traces of (a) SqTI-H (blue) with dashed line showing 3% mass loss attributed to removal of MeOH, (b) SqTI-tBu (violet) with blue dashed line showing ~25% mass loss possibly linked to formation of unsubstituted SqTI, (c) SqTI-iP (dark cyan) with dark cyan dashed line denoting ~20% mass loss predicted to correspond with formation of unsubstituted SqTI (not observed), and (d) SqTI-Hp (burgundy) with burgundy dashed line denoting ~37% mass loss predicted to correspond with formation of unsubstituted SqTI (not observed).

INFRARED (IR) SPECTROSCOPY

Solid state total reflectance ATR-IR spectra were obtained on an Agilent CARY 630 FTIR spectrometer.

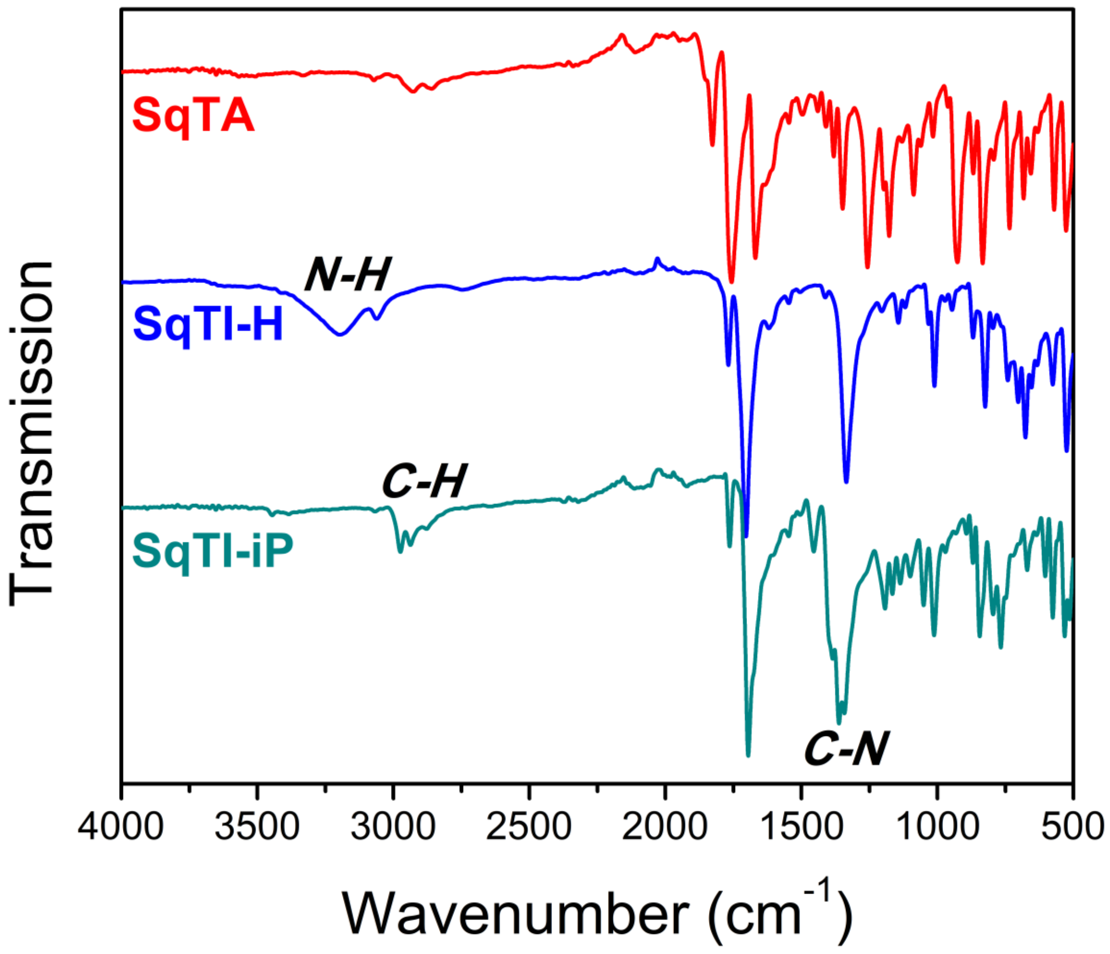

Figure S15: ATR-IR of SqTA (red), SqTI-H (blue) and SqTI-iP (dark cyan), with labelling of N-H stretching at 3200 cm^{-1} for SqTI-H, alkyl C-H stretching between 2800 – 3000 cm^{-1} for SqTI-iP as well as C-N stretching at 1340 cm^{-1} for both SqTI-H and SqTI-iP.

POWDER X-RAY DIFFRACTION (PXRD)

Powder X-ray diffraction (PXRD) data were collected with a Bruker D2 Phaser Diffractometer producing Cu-K_α (λ = 1.5418 Å) radiation, fitted with an SSD 160 detector.

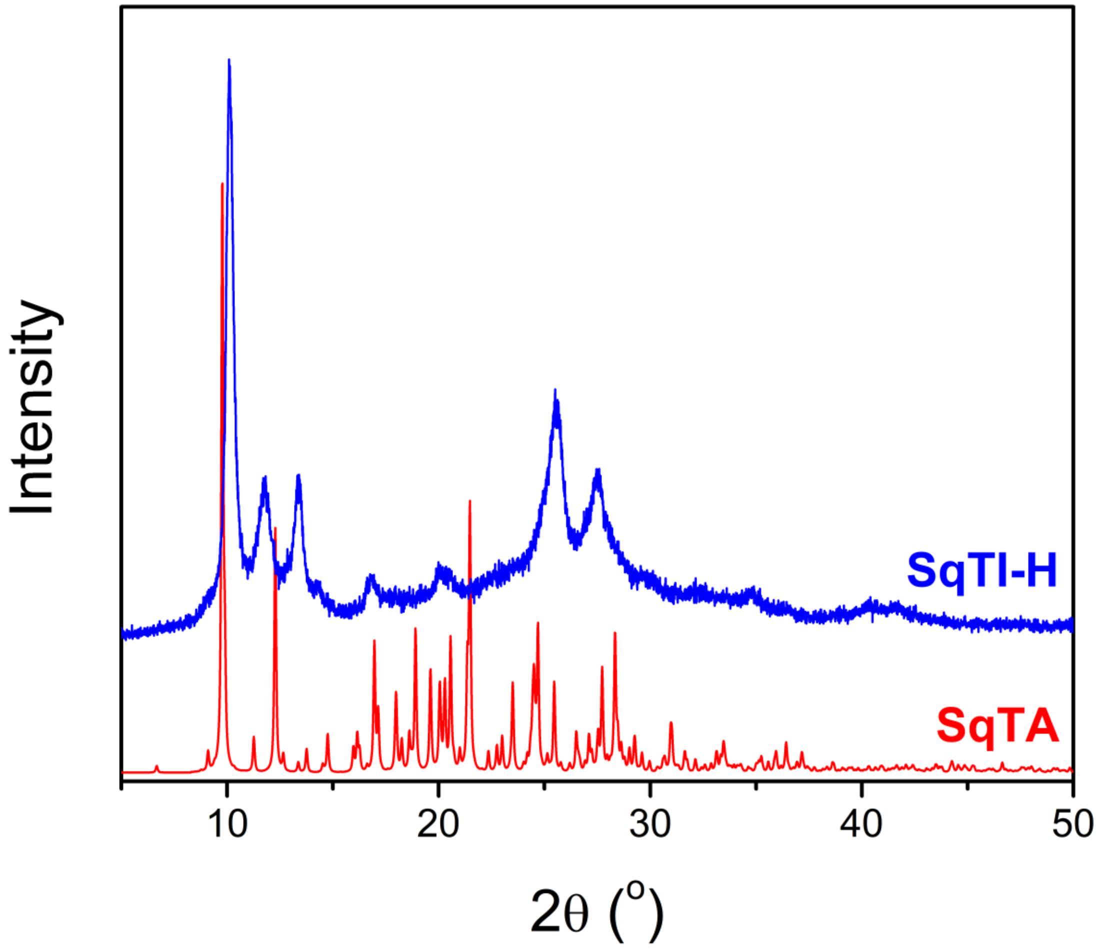

Figure S16: PXRD pattern of SqTI-H (blue), as compared to the predicted pattern of SqTA (red) from its single crystal X-ray diffraction structure.[1]

DIFFERENTIAL SCANNING CALORIMETRY (DSC)

Differential scanning calorimetry (DSC) traces between 40 – 300 °C were collected on a Mettler DSC822e differential scanning calorimeter, at a heating rate of 10 °C/min under a N_2 environment.

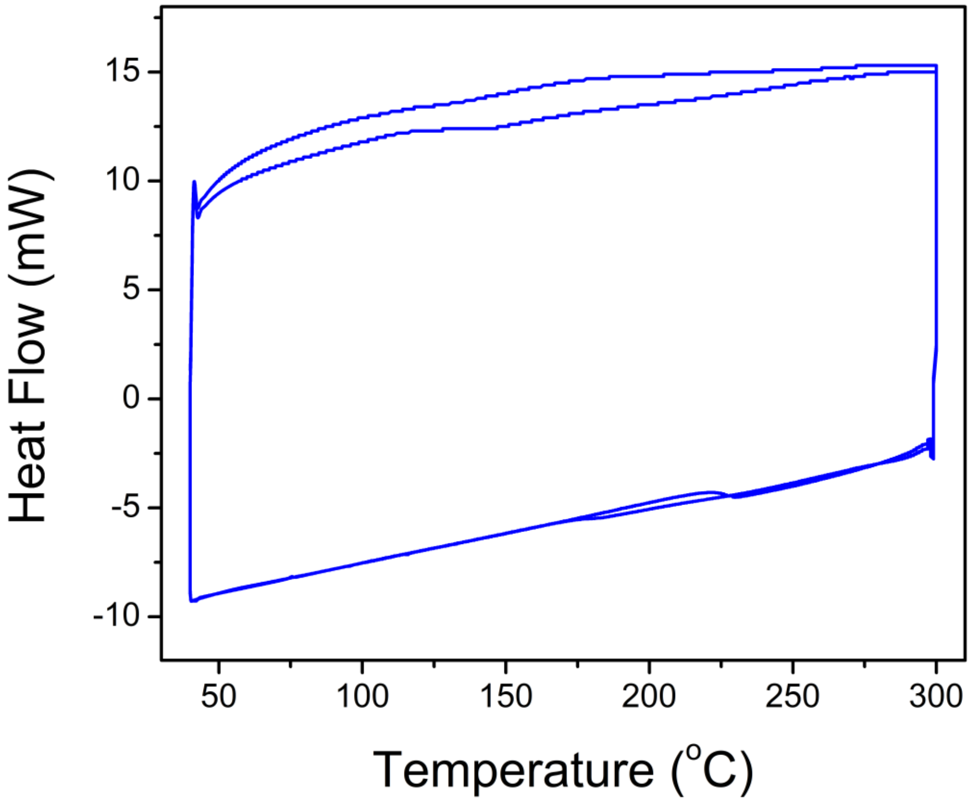

Figure S17: DSC trace (2 cycles) of SqTI-H between 40 to 300 °C.

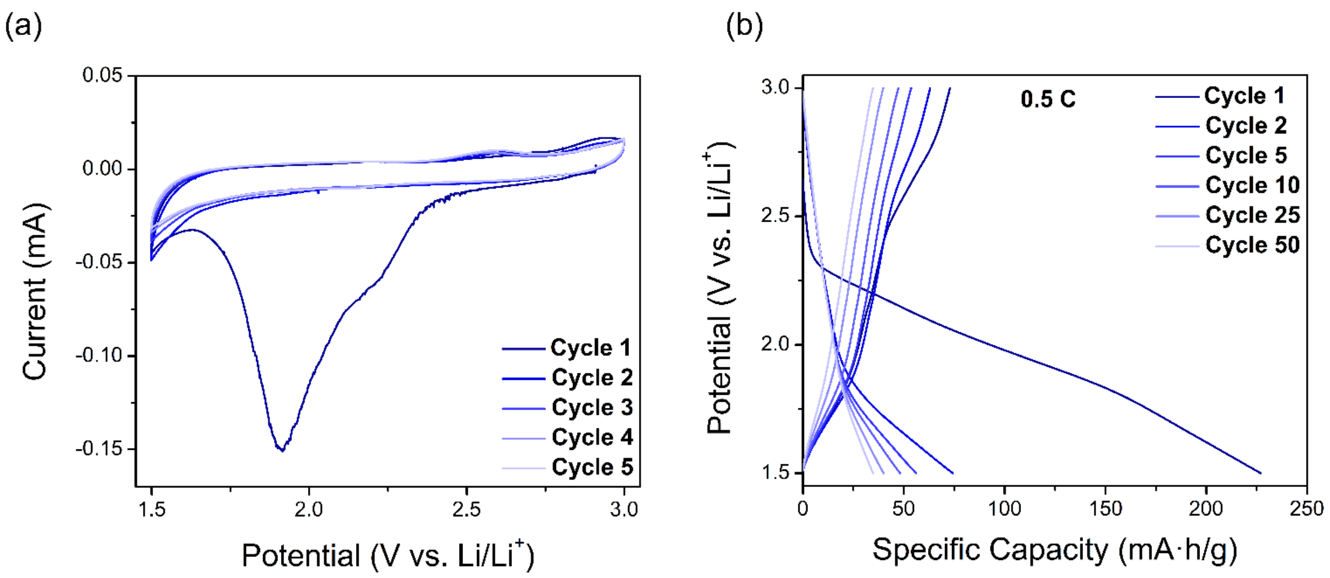

Figure S18: SqTI-H composite electrode performance in 1 M LiTFSI/1:1 1,3-dioxolane (DOL) and 1,2-dimethoxyethane (DME) showing (a) cycling voltammograms and (b) charge/discharge profiles at 50 mA/g (0.5 C) constant current cycling.

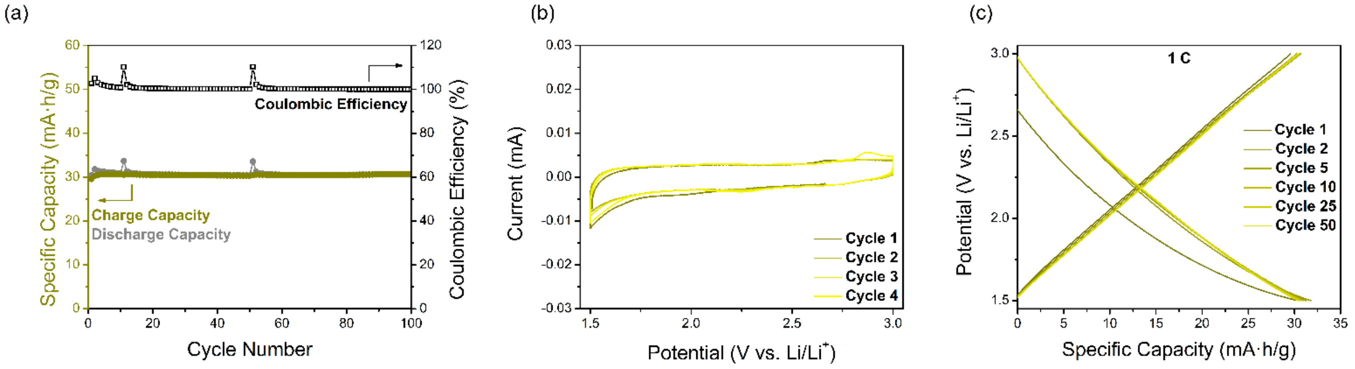

Figure S19: Ketjen Black electrode performance in 1 M LiTFSI/1:1 DOL+DME showing (a) cycling performance and coulombic efficiency, (b) cycling voltammograms and (c) charge/discharge profile at 100 mA/g (1C) constant current cycling.

REFERENCE

1. Eder, S.; Ding, B.; Thornton, D. B.; Sammut, D.; White, A. J. P.; Plasser, F.; Stephens, I. E. L.; Heeney, M.; Mezzavilla, S.; Glöcklhofer, F., *Angew. Chem. Int. Ed.* **2022,** *61*, e202212623.
